# Characterization and quantification of proteins secreted by single human embryos prior to implantation

**DOI:** 10.15252/emmm.201505344

**Published:** 2015-10-16

**Authors:** Maurizio Poli, Alessandro Ori, Tim Child, Souraya Jaroudi, Katharina Spath, Martin Beck, Dagan Wells

**Affiliations:** 1 Nuffield Department of Obstetrics and Gynaecology, Institute of Reproductive Sciences, University of OxfordOxford, UK; 2 Oxford Fertility Unit, Institute of Reproductive SciencesOxford, UK; 3 Reprogenetics UK, Institute of Reproductive SciencesOxford, UK; 4 European Molecular Biology Laboratory, Structural and Computational Biology UnitHeidelberg, Germany

**Keywords:** blastocoel, gene expression, human embryo, *in vitro* fertilization, proteomics

## Abstract

The use of *in vitro* fertilization (IVF) has revolutionized the treatment of infertility and is now responsible for 1–5% of all births in industrialized countries. During IVF, it is typical for patients to generate multiple embryos. However, only a small proportion of them possess the genetic and metabolic requirements needed in order to produce a healthy pregnancy. The identification of the embryo with the greatest developmental capacity represents a major challenge for fertility clinics. Current methods for the assessment of embryo competence are proven inefficient, and the inadvertent transfer of non-viable embryos is the principal reason why most IVF treatments (approximately two-thirds) end in failure. In this study, we investigate how the application of proteomic measurements could improve success rates in clinical embryology. We describe a procedure that allows the identification and quantification of proteins of embryonic origin, present in attomole concentrations in the blastocoel, the enclosed fluid-filled cavity that forms within 5-day-old human embryos. By using targeted proteomics, we demonstrate the feasibility of quantifying multiple proteins in samples derived from single blastocoels and that such measurements correlate with aspects of embryo viability, such as chromosomal (ploidy) status. This study illustrates the potential of high-sensitivity proteomics to measure clinically relevant biomarkers in minute samples and, more specifically, suggests that key aspects of embryo competence could be measured using a proteomic-based strategy, with negligible risk of harm to the living embryo. Our work paves the way for the development of “next-generation” embryo competence assessment strategies, based on functional proteomics.

## Introduction

Despite advances in assisted reproduction techniques over the last decade, it remains the case that only a minority of the embryos generated using *in vitro* fertilization (IVF) is capable of producing a viable pregnancy. In most IVF cycles, several embryos are produced. Maximal pregnancy rates then depend upon the identification of the most viable embryo, followed by transfer to the mother’s uterus. Unfortunately, current methods for distinguishing competent embryos from those that are incapable of producing a child are unreliable. The principal method of embryo evaluation, used in virtually all IVF clinics, is based upon morphological scoring. However, it is universally acknowledged that this approach is subjective and has only a limited ability to determine embryo potential (Machtinger & Racowsky, 2012).

The identification of reliable biomarkers of embryo development would lead to significant improvements in the efficiency of IVF treatment, increasing pregnancy rate per transfer, enhancing the cost-effectiveness of treatment, and eventually reducing patient’s emotional and financial stress. Additionally, there is growing pressure on IVF providers to minimize multiple pregnancy (e.g., twins, triplets, etc.) due to the increased risks of serious complications for the mother and babies and the impact on healthcare costs (Thurin *et al*, 2004). In order to reduce the likelihood of multiple gestation, a single embryo transfer policy is being adopted by an increasing number of clinics and enforced by healthcare governing bodies in several countries. However, if only one embryo is to be transferred, it is imperative that the embryo selected is the one with the greatest potential for producing a child.

For several years, researchers have been trying to identify molecules that correlate with embryo implantation competence, including metabolites, proteins, and reactive oxygen species. The methodologies used for these investigations, which ranged from Raman spectrometry to mass spectrometry and nuclear magnetic resonance, had different biochemical targets and varying degrees of sensitivity. So far, the vast majority of this research has focused on the analysis of the liquid in which single embryos were cultured, also described as conditioned media (Katz-Jaffe *et al*, 2006; Seli *et al*, 2008; Vergouw *et al*, 2008). One common problem of this approach is the vast excess of molecular components already present in the culture medium itself, which forms a complex background that can potentially mask key embryonic molecules present at extremely low concentrations (Dyrlund *et al*, 2014).

**Figure 1:**
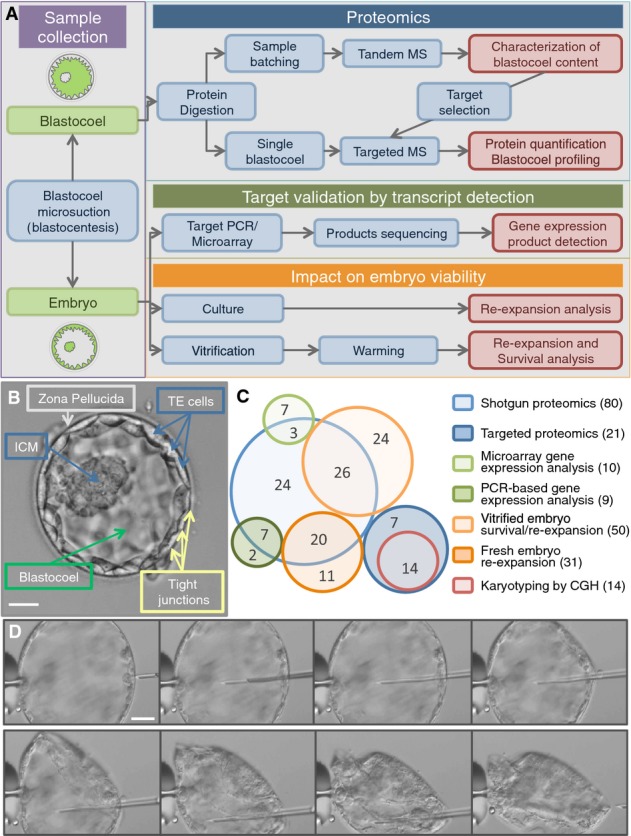
Investigating the blastocoel content using proteomics Study workflow and integration of proteomic, genomic, and embryology experiments.

Here, we describe the application of state-of-the-art proteomics technologies to clinical embryology, identifying and quantifying proteins secreted by the early human embryo in the hours before implantation (Fig1A).

The human embryo developed to the blastocyst stage. Five days after fertilization, a human blastocyst shows an inner cell mass (ICM) that will later develop into all the embryonic tissues and a trophectoderm (TE) that will form the extra-embryonic tissues (e.g., the placenta). TE cells are connected and held together by tight junctions that help to contain the fluid within the blastocoel cavity (blastosol). At this stage, the embryo is usually surrounded by a shell of oocyte-derived glycoproteins, the zona pellucida, from which it will “hatch” *prior to* implantation. Scale bar: 50 μm.

Embryo usage map. Each circle represents the embryo samples used in specific experimental set (blue, proteomics; orange, embryology; green, gene expression; and red, cytogenetics). Total number per technique is shown in brackets in the legend. In the circles, numbers correspond to the sample used for multiple experiments.

Sequence of photographs showing progression of the microsuction procedure (blastocentesis). Scale bar: 50 μm.

A normal human embryo grows into a fully formed blastocyst between 120 and 144 h post-insemination. By this time, the embryo has usually developed a full blastocoel, a fluid-filled cavity contained within a trophectoderm (TE) cell layer. The blastocoelic fluid, which here we define as blastosol, is also in contact with a group of cells called the inner cell mass (ICM), which are attached to the inner side of the TE layer, protruding into the cavity (Fig1B).

The blastocoel is a space where embryonic proteins are released and can accumulate. The transit of molecules in and out of the contained blastosol fluid is highly regulated (Watson *et al*, 2004). The blastosol is separated from the external environment by the surrounding monolayer of TE cells, which are linked together via tight junctions, thus forming an impervious barrier. Hence, the blastosol can provide a highly purified sample of embryo secretions, free from contaminants derived from the culture medium. In order to collect blastosol samples from viable embryos, we developed a micromanipulation technique that we named blastocentesis. This technique was previously employed by our team and other colleagues to source embryonic DNA for preimplantation genetic assessment, avoiding invasive biopsy procedures (Perloe *et al*, 2013; Poli *et al*, 2013; Gianaroli *et al*, 2014).

In this study, we investigate blastosol protein composition using an integrated proteomic, genomic, and embryological approach and using a total of 145 surplus human embryos donated by patients undertaking IVF treatment cycles (Fig1C).

We first examined blastosol composition by mechanically retrieving the fluid (∼5 nL) from multiple embryos (Fig1D), and analyzing pooled samples by tandem mass spectrometry. We were able to identify 288 proteins within the blastosol and to provide complimentary evidence of an embryonic origin for 182 of them by detecting the corresponding mRNA transcripts in whole embryos using a combination of micro-arrays and reverse transcription-PCR (RT–PCR). We then selected a subset of proteins of particular interest, based upon their abundance and their potential involvement in embryo implantation and development, and generated selected reaction monitoring (SRM) assays to enable analysis of blastosol samples from single embryos. We demonstrated that peptide detection and quantification can be achieved in blastocoels from individual embryos using targeted proteomics, despite the minute volume of the sample. We also show a potential correlation between the presence and abundance of target proteins in the blastosol with the chromosomal status of the whole embryo. Finally, this study demonstrates the applicability of proteomics technologies to biomarker discovery in general and in the field of human embryology in particular.

## Results

### Characterizing the blastocoel content

Blastosol samples were collected using blastocentesis, allowing the collection of 4–6 nL from each blastocyst cavity (Materials and Methods) (Movie EV1). Samples were initially processed using a standard procedure involving urea for protein solubilization and peptide desalting following enzymatic digestion. However, this approach was only compatible with pooled samples containing at least 20 blastocoels. For smaller samples, the majority of the peptides were lost during the multiple steps of the procedure. Therefore, a previously described nano-scale protocol was adapted for the analysis of individual blastocoel samples (Wang *et al*, 2005). This methodology utilized volatile buffers and organic solvents and had no requirement for peptide desalting prior to MS analysis (referred to as MonoPrep). In preliminary experiments, the MonoPrep procedure allowed the detection of more than 2,000 peptides comprising as little as 80 ng of proteins (cytoplasmic extract obtained from ∼1,000 HeLa cells) (FigEV1A). We performed shotgun proteomics analysis on 80 blastocoels sampled from human embryos 5 or 6 days after fertilization. The samples were divided into four sets each composed of 20 pooled specimens. Two sets were processed using the standard urea-based procedure and two with MonoPrep. For both procedures, blank samples obtained from plated sterile PBS microdrops swiped with a microneedle of the same type used for blastosol collection were analyzed in parallel and treated as controls for the identification of contaminant proteins.

**Figure EV1:**
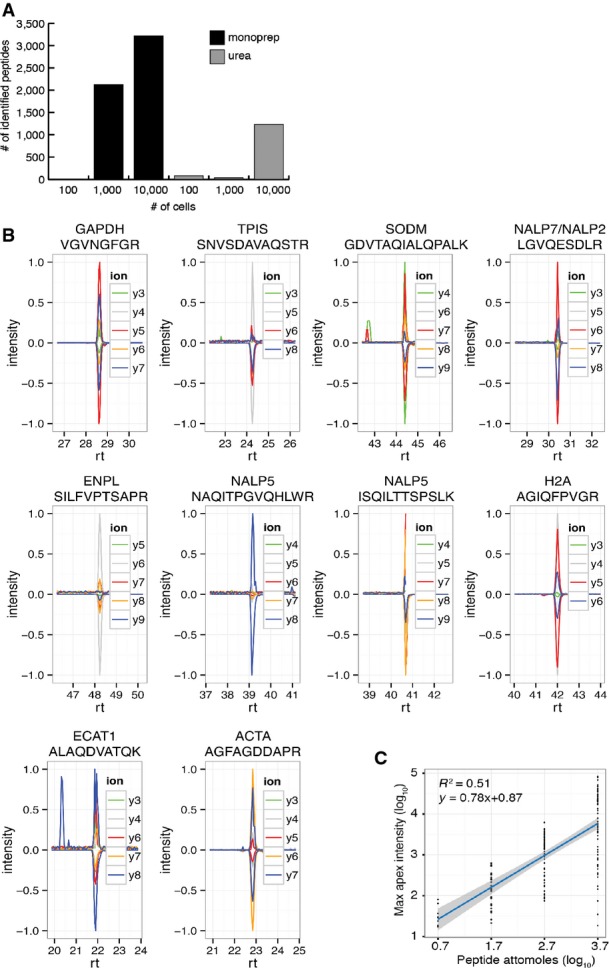
Comparison of proteomic workflows for blastocoel analysis and development of targeted proteomic assays for single embryo protein quantification Number of peptides identified using different sample preparation methods (black: solvent-based, MonoPrep; gray: urea-based, standard) on cytosolic lysates of HeLa cells at different concentrations (100, 1,000 and 10,000 cells corresponding to an estimated protein amount of 8, 80 and 800 ng, respectively).

Validation of ten SRM assays on samples of five blastocoels. Batched samples obtained from 5 blastocoels were spiked with isotopically labeled versions of the target peptides. SRM assays were manually validated on the basis of the co-elution and relative intensities of at least 4 transitions per assay. Transition traces are displayed using their relative intensities (with the intensity of the highest transition per assay being set to 1). Positive values are used for endogenous peptides, while negative values are used for reference (synthetic) peptides. Horizontal axis displays peptide retention time (rt) in minutes.

Intensity calibration curve derived from 76 absolutely quantified (AQUA) peptides measured at different concentrations. The linear regression between log_10_-transformed maximum apex intensity (derived from the maximum of the most intense transition trace) and log_10_-transformed attomoles of peptide injected was used to transform SRM-derived peptide intensities for blastocoel proteins into absolute abundances.

Single run data outputs from the samples were combined to have a comprehensive catalog of the fluid components. From the urea-based preparations, 169 proteins were identified, while 150 from MonoPrep samples, counting a total of 288 unique protein groups identified with a false discovery rate of 1% (Table EV1).

### Gene expression analysis in embryos

In order to provide additional verification of the embryonic origin of the proteins identified, gene expression analysis of human blastocysts was undertaken using microarrays. Cells forming the ICM were excised from the TE using a combination of micromanipulation and laser pulsing. Data generated by the individual samples were considered separately. Statistical analysis did not show significant difference in expression across any of the detected transcripts, and because of the high overlap between the expression catalogs (8,697 common transcripts 79.4%, FigEV2A), the results from the two groups were combined. This led to the creation of a catalog of 10,958 genes actively expressed in human blastocysts (provided in Table EV2).

**Figure EV2:**
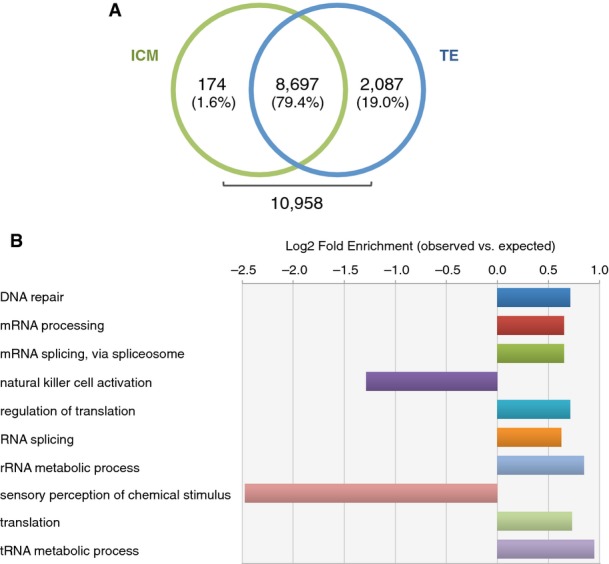
Transcript detection in individual embryonic tissues and graphical illustration of Panther statistical gene over-representation analysis in human blastocysts samples (Thomas *et al*, 2006; Mi *et al*, 2013) Actively transcribed genes in ICM (green) and TE (blue) cells. No transcript showed significant differential expression.

Blastocyst gene expression investigated using Panther database statistical overrepresentation test comparing global blastocyst transcript list to the default human whole-genome list, which included all genes present in the Panther database. Only biological processes of actively transcribed genes in the human blastocyst with fold enrichment < 0.5 and > 1.5 and *P*-value < 0.01 are shown. Bar lengths are displayed as Log_2_ of the ratio between observed and expected number of genes for each category. Raw data and *P*-values for each category are shown in Table EV3.

To provide a functional view of blastocyst gene expression profiles, we performed gene statistical over-representation analysis using Panther Classification System tools, based on GO-slim biological process annotation (Thomas *et al*, 2006; Mi *et al*, 2013). The majority of the biological activities showing significant enrichment (> 1.5 fold) compared to the reference human genome gene list involve both transcription and translation processes (FigEV2B and Table EV3). This increased number of active genes involved in tRNA and rRNA metabolic processes, combined with high transcription of genes required for regulation of mRNA maturation and translation, confirm an extremely active biogenesis activity in the developing blastocyst. Interestingly, genes involved in local immunological modulation (NK-cell activation and sensory perception of chemical stimulus) appear to be down-regulated, potentially reflecting the need to minimize any maternal inflammatory reaction to embryonic presence.

**Figure 2:**
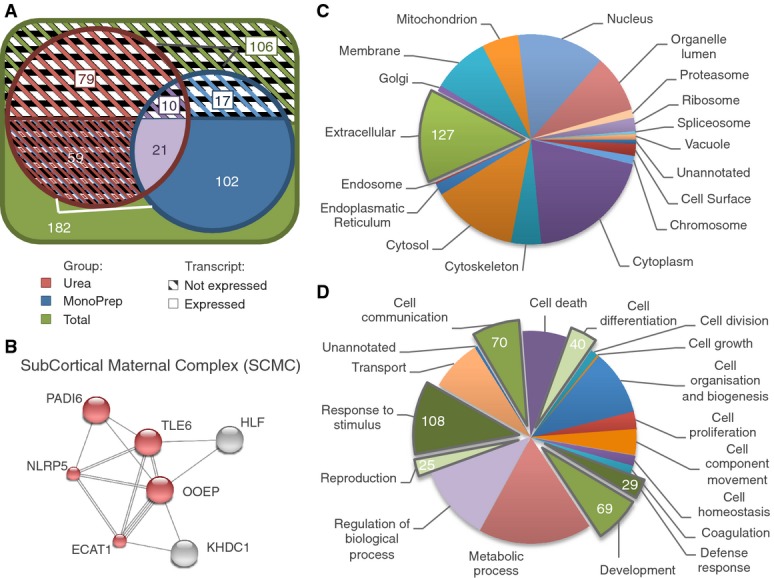
Proteomic characterization of the human blastocoel fluid A About 182 proteins identified from the blastocoel fluid had corresponding mRNA transcripts detected in embryos. The total number of proteins identified according to type of sample preparation method used is shown (red, urea; blue, MonoPrep; purple, shared; green, total). Full and barred sections refer to proteins confirmed and unconfirmed by gene expression of whole human blastocysts using microarrays, respectively.

The expressed transcripts were then cross-referenced (Materials and Methods) with the catalog of proteins identified using urea-based or MonoPrep tandem mass spectrometry blastosol analysis. Data show that 123 out of 150 (82.0%) proteins identified with Mono-Prep preparations were actively transcribed in the embryo, while for urea-based samples, only 80 out of 169 (47.3%) were confirmed by microarrays (Fig2A). Thirty-one proteins (10.8%) were identified in both urea and MonoPrep groups. Of these identifications, 21 (67.7%) were confirmed by gene expression analysis, while 10 (32.3%) were unconfirmed. A statistical analysis of protein abundance for each group showed that shared proteins were not generally more abundant than those identified by a single method (see Materials and Methods for details). Also, proteins confirmed by microarrays were not more represented in the shared group compared to the sum total of all proteins identified (chi-square test, *P*-value = 0.697). A potential explanation for the higher validation rate of the MonoPrep samples is that this procedure is less prone to the introduction of contaminant proteins such as keratins (Table1). However, it is possible that some of the detected proteins that could not be confirmed by gene expression analysis were coded by maternal RNA, originally produced in the oocyte. The proteins may have been synthesized prior to fertilization or during the first few days of life, but persisted to the blastocyst stage. To investigate this possibility, we cross-checked the list of identified proteins that were not confirmed by microarray with current literature on human oocyte transcriptome (Kocabas *et al*, 2006). Only one protein (out of 106) of potential maternal origin was revealed (PSMA7, Proteasome subunit alpha, type 7). This does not exclude the possibility that other proteins of maternal origin may persist in the cells of blastocyst stage embryos, but clearly any such proteins are absent from the blastocoelic fluid (or below the threshold of detection).

B The subcortical maternal complex (SCMC) is shown as an example of embryo-specific proteins identified. Complex subunits identified with mass spectrometry (MS) are shown in red, and protein interactions were derived from STRING (Jensen *et al*, 2009). Multiple lines indicate the existence of different evidences in the STRING database supporting the interaction.

C, D Gene Ontology (GO) classification indicating subcellular localization (C) and function (D) of the 182 blastocoel proteins identified by MS and validated with gene expression analysis. In (C), proteins localized in the extracellular space were highlighted as potentially involved in autocrine or paracrine signaling. In (D), groups with functions associated with embryo development and implantation processes were highlighted. GO annotations were obtained using Protein Center software (Thermo Fisher, USA). Note: Most proteins have multiple annotations.

**Table 1:** Most abundant and selected proteins identified from samples prepared with solvent-based, MonoPrep (top), and urea-based standard (bottom) methods

MONOPREP	Accession	Protein names	# peptides identified	Mw (KDa)	Abundance score (intensity/Mw)	Abundance rank	Expressed in Microarrays
10 most abundant proteins	P06454	Prothymosin alpha	5	12.191	1.24E + 04	1	✓
	P62805	Histone H4	3	11.354	1.04E + 04	2	✓
	P61604	10 kDa heat shock protein, mitochondrial (Hsp10)	4	10.919	6.00E + 03	3	✗
	P02766	Transthyretin (ATTR) (prealbumin) (TBPA)	2	15.869	4.02E + 03	5	✗
	P09936	Ubiquitin carboxyl-terminal hydrolase isozyme L1	4	24.796	2.94E + 03	6	✓
	P56279	T-cell leukemia/lymphoma protein 1A	2	13.444	2.89E + 03	7	✓
	Q14764	Major vault protein (MVP)	32	99.214	2.65E + 03	8	✓
	A6NGQ2	Oocyte-expressed protein homolog (OOEP)	5	17.15	2.21E + 03	9	✓
	Q08257	Quinone oxidoreductase	4	35.166	1.85E + 03	10	✓
	P04406	Glyceraldehyde-3-phosphate dehydrogenase (GAPDH)	4	36.012	1.67E + 03	11	✓
Selected proteins	P06454	Prothymosin alpha	5	12.191	1.24E + 04	1	✓
	A6NGQ2	Oocyte-expressed protein homolog (OOEP)	5	17.15	2.21E + 03	9	✓
	P04406	Glyceraldehyde-3-phosphate dehydrogenase (GAPDH)	4	36.012	1.67E + 03	11	✓
	Q587J8	KHDC3-like protein (ECAT1)	2	24.278	1.46E + 03	13	✓
	Q99497	Protein DJ-1	1	19.868	8.06E + 02	16	✓
	P00441	Superoxide dismutase [Cu-Zn]	1	15.918	7.46E + 02	18	✓
	P16949	Stathmin	2	17.283	4.05E + 02	27	✓
	P0DJG4	Testicular haploid expressed gene protein-like	1	52.968	3.80E + 02	31	*
	Q9H808	Transducin-like enhancer protein 6 (TLE6)	4	49.771	2.19E + 02	53	✓
	P14625	Endoplasmin (heat shock protein 90 kDa beta member 1)	6	92.365	1.90E + 02	56	✓
	P06576	ATP synthase subunit beta, mitochondrial	2	56.495	1.90E + 02	57	✓
	P04179	Superoxide dismutase [Mn], mitochondrial	1	24.694	1.26E + 02	72	✓
	P59047	NACHT, LRR, and PYD domains-containing protein 5 (Mater)	5	134.187	1.11E + 02	80	✓
	Q8WX94	NACHT, LRR, and PYD domains-containing protein 7	4	111.678	3.86E + 01	111	✓
	Q9NX02	NACHT, LRR, and PYD domains-containing protein 2	1	120.376	8.94E + 00	135	✓

UREA	Accession	Protein names	# peptides identified	Mw (KDa)	Abundance score (intensity/Mw)	Abundance rank	Expressed in Microarrays
10 most abundant proteins	Q01469	Fatty acid-binding protein, epidermal	9	15.147	9.24E + 05	5	✓
	P47929	Galectin-7	7	15.058	5.45E + 05	10	✗
	P22528	Cornifin-B	3	9.876	3.44E + 05	16	✓
	P10599	Thioredoxin	6	11.724	2.99E + 05	18	✓
	P01040	Cystatin-A	4	10.994	2.36E + 05	20	✗
	P04792	Heat shock protein beta-1	8	22.757	2.02E + 05	22	✓
	P55000	Secreted Ly-6/uPAR-related protein 1 (SLURP-1)	3	11.173	1.70E + 05	24	✗
	P15090	Fatty acid-binding protein, adipocyte	5	14.702	1.29E + 05	26	✗
	Q06830	Peroxiredoxin-1	6	22.085	9.51E + 04	28	✓
	P04406	Glyceraldehyde-3-phosphate dehydrogenase (GAPDH)	11	36.012	8.89E + 04	29	✓
Selected proteins	Q06830	Peroxiredoxin-1	6	22.085	9.51E + 04	28	✓
	P04406	Glyceraldehyde-3-phosphate dehydrogenase (GAPDH)	11	36.012	8.89E + 04	29	✓
	P06702	Protein S100-A9 (calgranulin B)	3	13.227	4.76E + 04	36	✓
	P05109	Protein S100-A8 (calgranulin A)	3	10.822	1.57E + 04	59	✓
	Q8WVV4	Protein POF1B (premature ovarian failure protein 1B)	13	67.989	1.34E + 04	61	✓
	P17931	Galectin-3	3	26.123	9.19E + 03	71	✓
	P14174	Macrophage migration inhibitory factor (MIF)	1	12.462	3.53E + 03	96	✓
	Q99497	Protein DJ-1	1	19.868	2.97E + 03	101	✓
	Q9NZH8	Interleukin-36 gamma (IL-1 epsilon)	1	18.7	2.78E + 03	104	✓
	P28799	Granulins	2	63.472	1.12E + 03	134	✓

Proteins identified in blank samples were considered as common contaminants and removed from the catalogue of identified proteins obtained from blastosol samples. The abundance ranking column refers to the relative protein intensity levels prior to exclusion of common contaminants. The proteins detected using the urea-based method show less continuous numbering of ‘abundance rank’ because a relatively large number of high abundance contaminants were detected and excluded.

* Gene not present in Illumina HT12_v4 Microarrays platform.

To validate microarray results, RNA was extracted from a batch of nine whole human blastocysts and reverse transcribed. Nested PCR was then carried out, seeking to amplify transcripts from four genes, the expression of which had been indicated by both microarrays (mRNA) and mass spectrometry analysis (protein). The genes assessed were as follows: granulins (GRA); oocyte-expressed protein (OOEP); NACHT, LRR, and PYD domains-containing protein 5 (NLRP5); and NACHT, LRR, and PYD domains-containing protein 7 (NLRP7). The detection of amplified DNA fragments of expected size and appropriate sequence confirmed the expression of all four genes (FigEV3 and Table EV4).

**Figure EV3:**
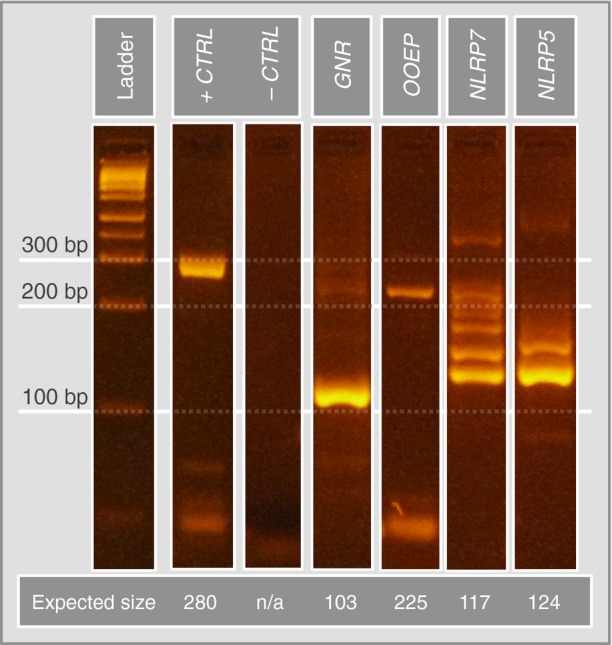
Validation of embryonic origin of selected MS-identified proteins using PCR Total mRNA was isolated from 9 whole blastocysts. Four target transcripts (granulins, GRN; oocyte-expressed protein, OOEP; NACHT, LRR, and PYD domains-containing proteins 5, NLRP 5; and NACHT, LRR, and PYD domains-containing proteins 7, NLRP 7) were amplified using nested PCR and custom-built primer sets. Expected bands were excised from the gel and subjected to Sanger sequencing to confirm the presence of the correct transcripts.

### Biological characteristics of blastosol components

By applying a combination of proteomic methods, the spectrum of known blastosol components was expanded, ultimately allowing the identification of 182 embryo-derived proteins. Newly identified proteins include six encoded by maternal-effect genes with specific expression at the zygote-embryonic stage, prior to activation of the embryonic genome, and correlated with key developmental functions (Zheng & Dean, 2009; Yurttas *et al*, 2010; Akoury *et al*, 2015). For example, we characterized the majority of the components of the previously described subcortical maternal complex (SCMC) (Li *et al*, 2008) including OOEP (also known as Floped), NLRP5 (also known as Mater), TLE6, PADI6, and ECAT1 (also known as Filia, KHDC3L, or C6orf221) (Fig2B). Contrary to previous reports (Herr *et al*, 2008; Ohsugi *et al*, 2008; Yurttas *et al*, 2010), we show that the components of this maternally inherited protein complex, crucial for oocyte to embryo transition, are still present both at transcript and at protein level after embryonic genome activation. Additionally, recurrent hydatiform moles have been linked to the presence of mutations in maternal-effect genes encoding for two proteins also identified in our experiments, NLRP7 and KHDC3L (Murdoch *et al*, 2006).

The biological function and cellular localization of the identified proteins were explored using Gene Ontology and are shown in Fig2C and D. In terms of localization, 127 proteins are annotated as confined and involved in functional processes in the extracellular region. Many of these proteins are engaged in the modulation of local immunogenic response and are either known or hypothesized to play roles in processes of response to stimuli, defense response, and cell communication. Some of the proteins that influence immune response might conceivably play roles in embryo implantation and communication between the blastocyst and the endometrium. For example, calgranulin A and B (also known as S100A8 and S100A9) have a pro-inflammatory function and their expression has been found to be significantly increased in products of early pregnancy loss (Nair *et al*, 2013). On the other hand, 173 proteins are annotated as localized in the cytoplasm where they take part in metabolic processes.

### Identification and quantification of selected targets in single blastocoels

**Figure 3:**
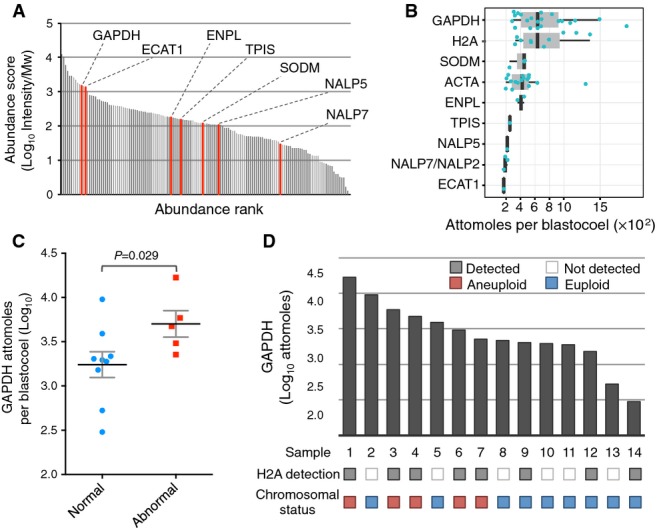
Targeted proteomics analysis of single human blastocoels Protein abundance profile of 20 pooled blastocoels. SRM assays were developed for the proteins shown in red.

In order to enable single blastocoel analysis, we developed selected reaction monitoring (SRM) assays for 10 peptides (corresponding to 9 protein groups) that were selected from among the most abundant peptides identified in the shotgun experiments (Fig3A). For assay development, spectral libraries generated from the discovery phase were used and employed a procedure previously described for peptide selection and assay development (Ori *et al*, 2014) (see Materials and Methods). Additional SRM assays aimed at identifying nuclear (H2A histone family) and cytoskeletal (ACTA) control targets were also developed. First, SRM assays using isotopically labeled synthetic peptides that were spiked in pools of blastosols obtained from 5 embryos were tested and successfully validated (Fig EV1B and Table EV5). Using these assays, 21 single blastosol samples were screened and the 9 tested proteins were detected, thus demonstrating the ability of targeted proteomics to measure multiple protein species deriving from a single embryo blastocoel (Tables EV6 and EV7). In order to estimate the concentration of the detected peptides in the blastosol, we first calibrated the signal intensity deriving from the mass spectrometer using a pool of absolutely quantified (AQUA) peptides derived from human proteins (Ori *et al*, 2013) that were injected in different known amounts (FigEV1C). The concentration of embryo-derived peptides was then estimated in the blastocoel fluid by linear regression using the fitted equation deriving from AQUA peptides. The concentration of the detected target proteins spans approximately one order of magnitude, ranging from 13 ng/μl (GAPDH) to 0.8 ng/μl (ECAT1) (Fig3B). For 16 blastocoels, multiple peptides were successfully detected (3.3 proteins per sample on average).

Summary of estimated protein abundances from measurements of 9 targets in 21 single blastocoel fluids. Absolute protein amounts were estimated from peptide intensities upon calibration using a set of absolutely quantified (AQUA) peptides (FigEV1B, see Materials and Methods for details). Blue dots correspond to individual data points. Black vertical bars indicate median values. Grey boxes show inter quartile ranges (IQR, Q3–Q1). Whiskers show Q1–1.5×IQR and Q3+1.5×IQR ranges.

GAPDH abundances per single blastocoel grouped based on embryo chromosomal status. *P*-values were calculated with Mann–Whitney *U*-test. Black bar: mean value; gray bars: SEM.

Measurements of GAPDH and detection of H2A family proteins in samples derived from 14 single blastocoels and chromosomal status of the embryo. The presence or absence of embryo aneuploidy was assessed by aCGH.

### Cytogenetic analysis of whole blastocysts

It was next considered whether variation in the protein content of individual blastocoels might provide useful information concerning embryo viability. Therefore, an attempt was made to correlate the proteomic profiles, derived from the intensities of the measured peptides, with embryo’s karyotype and other features including female patient’s age at time of treatment, embryo sex, and morphological score. In order to accomplish this, cytogenetic analysis of 14 fully expanded blastocysts, previously subjected to blastocentesis, was undertaken. The chromosomal status of each embryo was investigated using microarray comparative genomic hybridisation (array CGH) as described by Fragouli and colleagues (Fragouli *et al*, 2011), a highly validated method that provides comprehensive detection of aneuploidy in minute samples, including single cells. This test revealed that the group of embryos analyzed included seven euploid and six aneuploid embryos, seven of which were female, five male, and one Turner syndrome (45,XO).

To investigate the correlation between the presence and amount of the detected proteins and the physiological and genetic features of the embryo, we focused our analysis on proteins detected in the majority of samples (GAPDH, ACTA, and H2A protein family). The abundance of each protein was considered in relation to the embryo’s morphological score, karyotype, sex, female patient’s age at time of oocyte retrieval, semen parameters, and type of technique used for insemination (Table EV8). Individually, none of the protein targets showed significant differences across each embryo feature group (Table EV9). However, as shown in Table EV9, differences in GAPDH levels and H2A protein family detection across euploid/aneuploid groups produced *P*-values that, respectively, met and approached statistical significance following Mann–Whitney *U*-test and Fisher’s test (*P*-value = 0.029 and 0.056, respectively; Fig3C). In a subsequent logistic regression analysis, we used these two parameters as well as the ACTA levels as predictors and embryo’s ploidy status as class label (Fig3D). The statistical model was cross-validated with 3 folds and 20 repetitions. Within each cross-validation step, variable selection between the three predictors was performed using best subset selection with the AIC criterion (Akaike’s An Information Criterion). ACTA was only included in 7% of the cross-validation loops in the best model, while GAPDH levels and H2A protein family detection were included 92% and 97% of the time, respectively. This demonstrated that, together, the presence of H2A and abundance of GAPDH was able to predict whether an embryo was karyotypically normal or aneuploid. This was achieved with 100% accuracy within the cohort of samples investigated (see Materials and Methods for a detailed description of the analysis). It is worth noting that this accuracy estimate is likely to be overly optimistic due to the low sample size and the low number of predictors (3 in total) considered. Furthermore, validation in an independent series of samples would be necessary in order to obtain a true measure of diagnostic sensitivity and specificity.

### Effect of microsuction procedure (blastocentesis) on embryo viability

**Figure 4:**
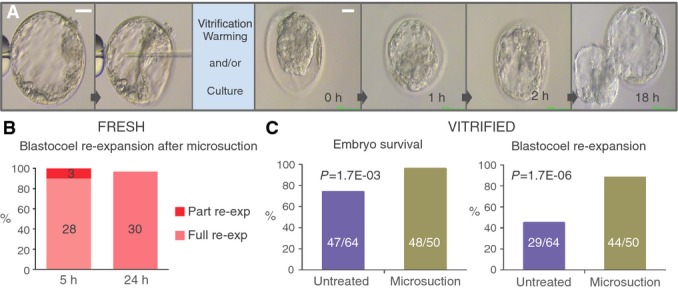
Blastocentesis is a safe procedure for the embryo, and it improves survival and blastocoel re-expansion following vitrification Time-lapse photography of a human blastocyst collapsed using microsuction prior to cryopreservation or culture (left) and subsequent re-expansion to normal morphology (right). Scale bars: 50 μm.

In order to evaluate the potential applicability of blastosol proteomic analysis to clinical samples, the impact of microsuction on embryo viability was assessed. The study focused on two sets of embryos that were subjected to blastocoel suction. The first group was composed of 31 blastocysts that were placed back in culture immediately after complete removal of the blastosol and monitored for the time required for the blastocoel to recover its original volume (Fig4A). All 31 embryos showed some degree of re-expansion within 5 h after collapsing (28 fully re-expanded (90.3%), 3 partially re-expanded (9.7%); Fig4B). At 24 h post-intervention, 30 (96.8%) embryos reached full re-expansion showing a uniform structural architecture and maintaining normal cell division, as demonstrated by their ability to hatch from the zona pellucida, the embryo’s outer shell (Fig4A).

Re-expansion rates of human blastocysts treated with microsuction prior to incubation for 5 and 24 h.

Embryo survival (left) and full re-expansion (right) rates in human blastocysts treated with microsuction or untreated prior to vitrification/warming and culture for 5 h. *P*-values calculated using two-tailed Fisher’s exact test (C.I. 95%).

The second group consisted of 50 blastocysts that underwent microsuction of the blastosol followed by cryopreservation using a well-established vitrification method. These embryos were later warmed and placed back in culture for 24 h, after which the survival rate and time to achieve full re-expansion were measured (Materials and Methods). The data collected were compared to the clinical outcomes achieved for embryos at the same developmental stage, cryopreserved using the same method, but without prior microsuction.

It was found that embryos in the experimental (microsuction) group showed higher post-warming survival rates compared to the control group (96.0% vs. 73.8%, respectively, *P*-value = 1.7E-03, two-tailed Fisher’s exact test, C.I. 95%) (Fig4C). This negative correlation between expansion of the blastocoel prior to vitrification and survival has also been described in published literature where blastocyst collapse was induced using alternative methods (Chen *et al*, 2005; Mukaida *et al*, 2006; Kader *et al*, 2010; e.g., laser pulse). In fact, the large inner water content of the blastocoel affects the diffusion of membrane-permeating cryoprotectants within the cavity, resulting in higher solution freezing point and increased risk of ice crystal formation. These crystals can damage cellular organelles, and nuclear and plasma membranes, resulting in cell lysis or induced apoptosis, often at lethal levels for the embryo (Vanderzwalmen *et al*, 2002; Kader *et al*, 2009).

In the experiments described here, embryos collapsed by microsuction also required shorter time to recover their original blastocoelic volume compared to the controls (88.0% vs. 44.6%, respectively, re-expanded within 4 h, *P*-value = 1.7E-06, two-tailed Fisher’s exact test, C.I. 95%) (Fig4C). It can therefore be concluded that microsuction does not have any detrimental effect on embryo viability and can in fact improve clinical embryo cryopreservation procedures. Clinical application of blastocentesis would simultaneously facilitate the beneficial collapse of the blastocoel cavity prior to vitrification and the collection of blastocoel fluid for downstream molecular analyses.

## Discussion

The identification of molecular markers of human embryo competence is an important topic in reproductive medicine. Novel effective embryo assessment procedures would maximize chances of treatment success and significantly reduce costs, leading to benefits to both patients and public healthcare systems. Here, we show that it is possible to detect and measure multiple embryo-derived proteins in single human blastocoels and that some of the proposed targets may provide valuable information regarding the developmental potential of the embryo. This study began with an extensive investigation of the content of the human blastocoel, providing a unique catalog of 182 embryo-derived proteins, subsequently validated by gene expression analysis. Seventy-six of these were described in a previous study, examining proteins contained in human blastocoels (Jensen *et al*, 2013). By applying a combination of sample preparation strategies, the most comprehensive blastocoel composition catalog was obtained. Additionally, by using a procedure that minimizes sample loss and contamination, we were able to achieve a greater degree of validation compared to conventional procedures, as judged by the detection of the corresponding protein-encoding transcripts in whole blastocysts. As expected, almost three quarters of the proteins identified have an extracellular location. Some of these proteins are known to be secreted by the cell and function as local immunogenic and developmental effectors, able to influence the implantation process, such as granulins (Díaz-Cueto *et al*, 2000) and microphage migration inhibitory factor (Bevilacqua *et al*, 2014). However, in line with previous studies (Jensen *et al*, 2013), proteins generally considered to have an intra-cellular localization were also detected, including heat shock proteins, metabolic enzymes, and components of the ribosome and proteasome. The majority of these proteins are known for their role in metabolic activity within the cell; however, it is likely that some of them are also involved in as yet uncharacterized extra-cellular processes. In contrast to secreted proteins, many of the intracellular proteins that we detected are expressed at high copy number within the cell. The difference in protein abundance between these groups can reach seven orders of magnitude, resulting in easier detection by MS of these intracellular components (Beck *et al*, 2011). A possible source of cytoplasmic proteins found in the blastosol could be traced to free-floating cellular fragments that, in certain circumstances, may be present in the blastocoel cavity and aspirated during microsuction (Movie EV2). The spontaneous formation of cellular fragments is a common feature of human embryos, is well documented in the literature, and is associated with reduced developmental competence (Fujimoto *et al*, 2011). Severe consequences of fragmentation on embryo viability include reduction in the number of cells comprising the embryo, extensive cytoplasmic depletion, and/or loss of genetic material from the cell due to incorporation into fragments. As a result, the quantity of cytoplasmic and nuclear proteins may serve as a potential marker to evaluate the extent of fragmentation damage (Mantikou *et al*, 2012). However, the possibility that the puncturing of the TE wall during the microsuction of the blastosol might cause some cells to rupture and release their contents into the cavity, leading to the aspiration of intracellular proteins, cannot be entirely excluded.

A key requirement for the application of proteomics for the assessment of blastosol composition is the ability to quantify proteins in specimens derived from individual embryos. With this aim, a preparation technique that enabled the simultaneous detection of nine protein targets of interest was developed and successfully applied to 21 individual human blastocoels.

A major challenge of this methodology is the sensitivity required to detect proteins present in the minute blastosol volume. In fact, among the proteins tested, only those with higher abundance were regularly detected in the specimen (Fig3B). This limited sensitivity could be attributed both to technical and biological aspects. As shown in Fig3A, some of the proteins that were detected only in a subset of samples show lower than average concentrations, as determined from their intensities in shotgun proteomic experiments. The reproducible quantification of these targets is more challenging since their concentration is close to the limit of detection of our targeted proteomic setup. However, the inconsistent detection of some targets, as in the case of histones proteins (H2A), could be attributed to a biological variability, possibly associated with deviant physiological conditions. Increased detectability of targets should be addressed in the future by targeting alternative peptides, further optimizing procedures to minimize sample loss and utilizing alternative investigative devices, including both more sensitive mass spectrometers and digital immunoassays.

Nonetheless, the SRM assays utilized in the study generated valuable data for those proteins with higher abundance. The data obtained suggested that blastocoels of euploid embryos tend to show lower abundance of GAPDH (Fig3C). This protein is an intermediate enzyme of the glycolysis pathway, the first in a series of catabolic reactions that produce ATP. It has previously been suggested that embryos displaying increased metabolic rate and energy consumption are associated with lower developmental potential (Leese, 2002). GAPDH shows both intra- and extracellular localization; therefore, the precise origin of the protein detected in blastosol samples cannot be determined with certainty. However, given that the detected abundance of actin, an entirely intracellular cytoskeletal component, was not significantly different across the samples tested, it seems likely that the observed variation in GAPDH levels was not related to differences in the amount of cellular fragments present in the blastocoel. Interestingly, histone H2A family members were detected more frequently in blastosol samples from aneuploid embryos, suggesting that cell-free DNA fragments may be more common in embryos with an abnormal karyotype. In this circumstance, the detection of chromatin-associated proteins may act as direct evidence of aberrant chromosome segregation. The presence of proteins, typically considered to have a nuclear localization, in the blastocoel cavity, may be indicative of release following cell lysis, possibly related to apoptotic processes.

The application of logistic regression analysis to the data obtained from blastosol protein profiling and aCGH suggested that it may be possible to distinguish chromosomally normal embryos from those affected by aneuploidy, based upon the levels of GAPDH and the detection of histone H2A proteins in the blastocoel fluid. In this initial cohort of samples, embryos that were aneuploid were identified with 100% accuracy. Together, the measurement of GAPDH and identification of H2A family proteins may provide a summary of the metabolic and genetic status of the embryo.

However, due to the small size of the sample population investigated, the limited amount of predictors used, and the retrospective nature of the analysis, these data need to be treated cautiously. It is essential to generate additional data in larger studies in order to test the validity of this proteomic approach to preimplantation aneuploidy detection and to define the real sensitivity and specificity of this methodology. Also, targeted mass spectrometry represented the most sensitive technology that allowed us to design assays for a number of target proteins without the need for specific reagents (e.g., antibodies). In the future, alternative novel methodologies (i.e., single molecule arrays, digital ELISA) could be implemented to improve the sensitivity and comprehensiveness of human blastosol profiling in single embryos.

Finally, for blastocentesis to be applied as a clinical tool, the microsuction procedure must be proven safe to the embryo. Blastocoel collapse is a common process, often applied to *in vitro* cultured blastocysts in order to improve embryo survival during cryopreservation procedures (Vanderzwalmen *et al*, 2002; Mukaida *et al*, 2006; Hur *et al*, 2011; Iwayama *et al*, 2011). Observation of treated embryos suggests that blastosol composition is quickly re-established by viable embryos. In this study, we show that, similar to other routine methods used to collapse the blastocyst, blastocentesis does not affect embryo overall architectural structure. Hence, blastocentesis offers a safe mean to the collection of blastosol samples for proteomic or genomic analysis, while effectively reducing embryo volume prior to cryopreservation. However, while any negative impact seems unlikely, long-term effects of this procedure on embryo functionality and pregnancy should be assessed in a clinical trial.

On a technical note, blastocentesis does not require skills other than those routinely practiced in all IVF clinics. Furthermore, the equipment required is standard in all embryology laboratories. Since no additional equipment or training is required, it is conceivable that blastocentesis could be rapidly implemented in IVF laboratories worldwide. This technique not only allows a cost-effective method for blastocoel collapsing prior to cryopreservation, but also enables the collection of a potentially meaningful embryo-derived sample, which would otherwise be discarded.

In conclusion, a “next-generation” methodology able to assess embryo competence, improving IVF success rates and reducing the likelihood of multiple pregnancies, is urgently required. Here, we demonstrate an exquisitely sensitive method of proteomic analysis, capable of detecting and quantifying specific proteins in nanoliter volumes of fluid derived from the blastocoel cavity of human preimplantation embryos. It is likely that this functional proteomic approach will yield novel biomarkers for the evaluation of embryo potential and could conceivably be applied in other, diverse areas of clinical research involving single cells, microscopic samples of tissue, or minute volumes of biological fluid.

## Materials and Methods

### Ethical approval

This study was integrated in an existing research study approved by the NRES Committee South Central – Oxford (Ref. 04/Q1606/44) and licensed by the Human Fertility and Embryology Authority (R0111). Patients were enrolled in this study following HFEA guidelines on surplus embryos donation to research.

### Embryology

#### Production of human embryos

Embryos used in this study were donated by couples undergoing IVF treatment at the Oxford Fertility Unit, UK. All patients underwent conventional controlled ovarian hyperstimulation, and oocyte collection was performed following follicle triggering by 10,000 IU recombinant hCG (Ovitrelle, Merck Serono, Germany). Collected oocytes were inseminated by exposure to fixed concentration of partner’s treated sperm sample or by intracytoplasmic sperm injection (ICSI). Fresh embryos were individually cultured in 50 μl microdrops of Cleavage medium (COOK, Australia) up until day 3 post-fertilization and then moved to 50 μl microdrops of Blastocyst medium (COOK, Australia) until donation. Donated cleavage-stage frozen embryos were thawed using standard commercial kit (COOK thawing media) and then cultured to blastocyst stage using Blastocyst medium (COOK, Australia). Incubation parameters were 37°C and 6% CO_2_ and 5% O_2_.

Fresh embryos were donated on day 5 or 6 post-insemination, while frozen embryos were donated at various stages between 24 and 72 h post-insemination. Frozen embryos were thawed and cultured to day 5 or 6 until a full cavity was present.

#### Blastocoel collection

Blastocysts were individually washed in 1 mL PBS to remove proteins present in the culture media prior to placing them into a microsuction setup dish.

Blastocoel extraction procedures were performed in 10 μl drops of sterile micro-filtered PBS, overlaid with sterile mineral oil (COOK, Australia). The micromanipulation technique used involved anchoring the embryo to a holding pipette (Research Instruments, UK) on one side and inserting a microinjection needle (Research Instruments, UK) through the trophectoderm cell layer on the opposite side. Gentle application of negative pressure on the needle allowed fluid collection and embryo collapse (Fig1C and Movie EV1). The aspirated fluid was expelled in an adjacent 3 μl microfiltered PBS supplemented with 3 mM ammonium bicarbonate drop (Sigma-Aldrich Co. LLC, USA) and then collected in a 0.2-ml PCR tube using a sterile 130-μm-diameter Flexipet (Research Instruments, UK).

#### Blastocyst vitrification

Blastocyst vitrification was performed using Cook Blastocyst Vitrification media kit (Cook, Australia) and CVM vitrification system (Cryologic, Australia).

As recommended by the manufacturer, the embryo was washed in Solution 1 at 37°C for 30 s and then moved into Solution 2 at 37°C for 2 min. The embryo was then moved to Vitrification Solution 3. Within 20–30 s, the embryo was loaded on the Cryohook (Cryologic, Australia). The loading device was then placed onto the vitrification block allowing the droplet containing the embryo to vitrify immediately. The vitrified sample was then inserted into a straw and firmly secured by hand. Finally, the straws were transferred to a permanent storage dewar.

#### Blastocyst warming

Blastocyst warming was performed using Cook Blastocyst Warming media kit (Cook, Australia). The loading device was removed from the outer straw and directly submerged into Solution 1 at 37°C. The embryo was immediately moved to a second well containing Warming Solution 1 and incubated at 37°C for 5 min. This procedure was repeated for Solutions 2 and 3. Finally, the embryo was placed in a culture dish containing Cook Blastocyst Culture media (Cook, Australia) at standard culture conditions for a period between 3 and 18 h *prior to* discarding.

#### Blastocyst morphological assessment

Parameters evaluated included degree of expansion, ICM morphology, and TE morphology, according to Gardner’s blastocyst assessment criteria (Gardner & Schoolcraft, 1998; Gardner & Leese, 1999). Embryos that scored grade A or B in ICM or TE assessment were considered of *good* morphology unless they scored D or E for the other parameter. Embryos that showed ICM or TE of grade C were considered *good* if the other parameter was an A or a B, and *poor* if the other parameter was a C, D, or E.

#### Blastocyst survival assessment

Post-warming embryo survival assessment was performed using clinical practice criteria. Embryos were evaluated with microscopic analysis and were considered fully survived if > 50% of cells were found intact. Cells were regarded as viable if plasma membranes showed a clear margin and a homogenous content. If membranes were found blurred with dark, granular content, cells were considered dead.

#### Blastocyst re-expansion assessment

After thawing, blastocysts were cultured and assessed for expansion at regular intervals. Expansion was graded as *absent* if < 10% of the original volume was recovered, *partial* if around 50% was recovered, or *full* if around 100% of expansion was achieved.

### Proteomics

#### Protein digestion and peptide desalting for urea-based procedure

A 5 μl solution of 10 M urea and 125 mM ammonium bicarbonate was added to each sample tube immediately after collection. Samples were then stored at −80°C. At time of processing, the tubes were thawed and spun at 13,000 *g* for 5 min. Supernatants from 20 samples were batched and transferred to a fresh tube, and dithiothreitol was added to reach 10 mM and incubated 30 min at 37°C with mixing (800 rpm). Iodoacetamide was added to reach 15 mM and incubated 30 min in the dark. LysC (Wako) was added to an estimated enzyme:protein ratio of 1:100 (w/w) and incubated for 4 h at 37°C. The urea concentration was then diluted to 1.5 M using HPLC-grade water, trypsin (Promega) was added to reach an estimated enzyme:protein ratio of 1:50 (w/w), and samples incubated overnight at 37°C. Samples were acidified with 0.5% (v/v) trifluoroacetic acid (TFA) to stop digestion and spun at 13,000 *g* for 5 min. The supernatants were moved to a fresh tube and desalted using C18 spin columns (Ultra-micro spin columns, Harvard Apparatus) according to the manufacturer’s instructions.

#### Protein digestion for MonoPrep procedure

Acetonitrile was added to the sample to reach a concentration of 40% (v/v). About 1 μl of diluted trypsin (0.01 μg/μl) was added to reach an estimated protein:enzyme ratio of 1 (w/w). Samples were incubated at 37°C overnight and then acidified with 0.5% (v/v) TFA to stop digestion. Digested samples were stored at −80°C until MS analysis.

#### Shotgun mass spectrometry

Samples were analyzed using a nanoAcquity UPLC system (Waters GmbH) connected online to a LTQ-Orbitrap Velos Pro instrument (Thermo Fisher Scientific GmbH) as described by Ori *et al* (1999) Briefly, peptides were separated on a BEH300 C18 (75 μm × 250 mm, 1.7 μm) nanoAcquity UPLC column (Waters GmbH) using a stepwise 145 min gradient between 3 and 85% (v/v) ACN in 0.1% (v/v) FA. Data acquisition was performed in data-dependent mode using a TOP-20 strategy where survey MS scans (m/z range 375–1,600) were acquired in the orbitrap (*R* = 30,000 FWHM) and up to 20 of the most abundant ions per full scan were fragmented by collision-induced dissociation (normalized collision energy = 35, activation *Q* = 0.250) and analyzed in the LTQ. Ion target values were 1,000,000 (or 500 ms maximum fill time) for full scans and 10,000 (or 50 ms maximum fill time) for MS/MS scans. Charge states 1 and unknown were rejected. Dynamic exclusion was enabled with repeat count = 1, exclusion duration = 60 s, list size = 500, and mass window ±  15 ppm. Raw files were analyzed using MaxQuant (version 1.2.2.5) (Cox & Mann, 2008). For urea-based procedure, samples were analyzed in technical duplicate (repeated injection of the same sample) and the results combined. MS/MS spectra were searched against the human Swiss-Prot entries of the UniProt release 2011_12 using the Andromeda search engine (Cox *et al*, 2011). The search criteria were set as follows: full tryptic specificity was required (cleavage after lysine or arginine residues, unless followed by proline); 2 missed cleavages were allowed; carbamidomethylation (C) was set as fixed modification (only for samples from urea-based procedure); oxidation (M) and acetylation (protein N-term) were applied as variable modifications, if applicable; and mass tolerance of 20 ppm (precursor) and 0.5 Da (fragments). The reversed sequences of the target database were used as decoy database. Peptide and protein hits were filtered at a false discovery rate of 1% using a target-decoy strategy (Elias & Gygi, 2007). The “peptides.txt” output file of the MaxQuant search was used to calculate protein abundance scores from the summed intensities of proteotypic (unique) peptides normalized by the protein molecular weight, as described by Ori and colleagues (Ori *et al*, 2013). Proteins were assigned as deriving from the blastocoel if they were identified by at least one proteotypic peptide and they were not detected in blank samples. Mass spectrometry proteomics data were deposited to the ProteomeXchange Consortium (http://proteomecentral.proteomexchange.org) (J. Vizcaíno *et al*, 2014) via the PRIDE partner repository (Vizcaíno *et al*, 2013) with the dataset identifier PDX002566.

### Targeted mass spectrometry

SRM assay development, validation, and peptide quantification were performed using SpectroDive (a kind gift of Biognosys AG). Ten target peptides were selected among the most intense peptides identified in the shotgun experiments (only MonoPrep samples were considered). SRM assays were developed and validated as described by Ori and colleagues (Ori *et al*, 2013). Briefly, isotopically labeled version of the selected peptides were purchased from JPT Peptides Technologies GmbH, mixed, and spiked into pooled samples of blastocoels derived from 5 embryos. SRM assays were validated by the detection of co-eluting transitions from the endogenous (light) and standard (heavy) peptides (FigEV1B and Table EV5). For the analysis of single blastocoels, we decided not to spike-in reference heavy peptides to avoid interference with the low intensity signal deriving from the endogenous peptide. However, we manually inspected that both retention time and relative order of transitions were consistent with the validated assays. For sample comparison, peptide intensities were derived from the summed intensity of the transitions. In order to calibrate signal intensities to absolute peptide concentrations, we used a pool of 76 AQUA peptides that were previously used to determine the stoichiometry of the human nuclear pore complex (Ori *et al*, 2013). AQUA peptides were injected in four different concentrations (5, 50, 500, and 5,000 attomoles), and signal intensities (max apex) were fitted by linear regression (FigEV1C).

### Gene expression analysis

#### Microarrays

Data were obtained from a total of 10 embryos. For each embryo, ICM and TE were separated and distributed into three paired replicates (three ICM and three TE). Fully expanded human blastocysts were placed in 10 μl microdrops of filtered PBS. Embryos were anchored to a holding pipette (Cook, Australia), while part of the zona pellucida corresponding to the ICM was removed by laser pulse (Research Instruments, UK). Using a 35-μm Blastomere aspiration pipette (Cook, Australia), gentle aspiration was applied and the portion containing the ICM was extracted from the zona pellucida. At need, small laser pulses were applied to the TE to facilitate the detachment of the biopsied sample from the rest of the embryo. Biopsied cells were individually transferred to sterile PCR tubes in 2.5 μl of PBS solution and kept at 4°C to minimize cellular degradation before storing at −80°C. Two sample pairs were composed of pooled cellular material from three and one pair from four blastocysts. Each replicate was analyzed separately, and the list of active genes was combined. Embryos used in these experiments were day 5/6 embryos deriving from fresh treatment cycles or embryos that were thawed at day 3 of development and then cultured to blastocyst stage. The mRNA from the samples was isolated using RNaqueous-Micro Kit (Life Technologies) and following the manufacturer’s instructions. Briefly, after ethanol-based cell lysis, cellular content was filtered using a micro filter cartridge by centrifugation. The filtered products were washed with commercial solution prior to elution. RNA was then collected and treated for DNA digestion. The RNA was collected with the supernatant and placed in a new RNAse-free PCR tube.

Purified mRNA was amplified using TargetAmp 2-Round Biotin-aRNA Amplification Kit 3.0 (Epicentre, Illumina) following the manufacturer’s instructions. Briefly, this kit allows two subsequent *in vitro* transcription reactions and the incorporation of biotin-labeled nucleotides in the last step, starting from purified RNA. As suggested by the manufacturer, SuperScript III and SuperScript II Reverse Transcriptase (Invitrogen, Life Technologies, USA) were used for 1st strand cDNA synthesis in the first and second amplification rounds. RNA Clean & Concentrator-5 Kit (Zymo Research) was used for RNA purification after the first round of *in vitro* transcription. Final biotin-aRNA purification was performed using RNeasy MinElute Cleanup Kit (Qiagen, Germany). No freezing step was performed during the procedure to avoid sample degradation.

Total RNA output was measured by spectrophotometry analysis using a Nanodrop device (Thermo Fisher, USA). Aliquots of all samples were normalized to 150 ng/μl to allow microarray hybridization. Sample hybridization to HumanHT-12 v3 Expression BeadChip microarrays (Illumina, USA) was performed according to the manufacturer’s protocol.

Raw data were imported into the R statistical software (R Development Core Team, 2013) for further processing and analysis using BioConductor packages (Gentleman *et al*, 2004). Raw signal intensities were background corrected prior to being transformed and normalized using the “*vsn*” package (Huber *et al*, 2002). The background intensity range was defined by a set of several hundred negative control probes on the array, designed with no match in the human genome. Probes were assigned a detection *P*-value, based on their signal overlap with the background distribution. A detection *P*-value < 0.05 was used to define probes expressed above background levels. Probes with signals above background levels in all of the samples were classified as expressed. These were annotated with gene information using the relevant BioConductor annotation package (Dunning M, Lynch A and Eldridge M. *illuminaHumanv4.db: Illumina HumanHT12v4 annotation data (chip illuminaHumanv4)*. R package version 1.22.1.).

The genomic data discussed in this publication have been deposited in NCBI’s Gene Expression Omnibus (Edgar & Lash, 2002) and are accessible through GEO SuperSeries accession number GSE72685 (http://www.ncbi.nlm.nih.gov/geo/query/acc.cgi?acc=GSE72685). Human blastocyst gene expression data are directly accessible through the GEO SubSeries accession number GSE71455 (http://www.ncbi.nlm.nih.gov/geo/query/acc.cgi?acc=GSE71455).

#### Target PCR and product sequencing

Embryos were washed in clean PBS and transferred in a sterile PCR tube in a volume of 1.5 μl. RNA from 9 batched blastocysts was purified and converted to cDNA using a commercially available kit “Cells to cDNA II Kit” (Ambion, Life Technologies). The protocol was employed as described by the manufacturer, apart from the cell lysis step, where a reduced volume of lysis buffer was used to increase RNA final concentration. The optional step for DNA digestion was also employed as described by the manufacturer. The cDNA generated by this protocol was split into several reactions aimed at amplifying different outer products of interest.

Nested PCR amplification was used to detect products of interest with maximum sensitivity. Primers for granulins (*GRA*); oocyte-expressed protein (*OOEP*); and NACHT, LRR, and PYD domains-containing protein 5 and 7 (*NLRP5* or *MATER* and *NLRP7*) were custom designed using PrimerBLAST, Oligocalc, and Primer3 softwares. All primers were verified using GenBank BLAST Primer database for sequence specificity, primer dimer, and loop formation (Table EV4).

Tm for both inner and outer primers were empirically validated by amplifying genomic DNA on a gradient PCR. Elongation step times for each PCR were set according to the expected product size.

Products from the outer and inner reactions were plotted on 3% agarose gel for 80 min at 80 mV.

Gel bands of interest were excised, and DNA was purified according to Qiagen DNA gel extraction kit (Qiagen, Dusseldorf, Germany). Aliquots of the purified PCR products and primers were sent for Sanger sequencing to a commercial service provider (Source BioScience, Oxford). The origin of the mRNA sequence was then validated using BLAST database annotation homology.

### Cytogenetics

#### Array CGH

Whole-genome amplification was carried out using SurePlex (Illumina, USA) according to the manufacturer’s instructions. Amplified DNA from the embryo and previously amplified normal male (46,XY) DNA (SureRef; Illumina, USA) were fluorescently labeled with the use of the Fluorescence Labelling System (Illumina, USA). “Test” embryonic DNA was labeled in Cy3, and the “reference” 46,XY DNA was labeled with Cy5. Test and reference DNA co-precipitation, denaturation, array hybridization, and post-hybridization washes took place as recommended by the manufacturer. Probes hybridization was performed over 16 hrs. A laser scanner (InnoScan 710, Innopsys, France) was used to excite the hybridized fluorophores, and to read and store the resulting images of the hybridization. MAPIX software (Innopsys, France) was used to control the scanning of the microarray slides. The resulting images were stored in TIFF format file and analyzed by the BlueFuse Multi v3.1 analysis software (Illumina, USA). Chromosome profiles were be examined for chromosomal gain or loss with the use of a 3 × SD assessment, identical to the analysis performed for clinical trophectoderm samples. Embryos were classified as euploid or aneuploid, and female or male according to this assessment.

The genomic data discussed in this publication have been deposited in NCBI’s Gene Expression Omnibus (Edgar & Lash, 2002) and are accessible through GEO SuperSeries accession number GSE72685 (http://www.ncbi.nlm.nih.gov/geo/query/acc.cgi?acc=GSE72685). Embryo cytogenetics data are directly accessible through the GEO SubSeries accession number GSE72684 (http://www.ncbi.nlm.nih.gov/geo/query/acc.cgi?acc=GSE72684).

### Statistics

Protein abundance distribution per identification group (confirmed urea only vs. confirmed urea Shared; confirmed MonoPrep only vs. confirmed MonoPrep shared) was assessed using SPSS software. All groups showed normal distributions. Statistical analyses of protein abundance across identification groups were performed using *t*-test on GraphPad software. The analysis showed no significant difference between the groups analyzed (urea only vs. urea shared, *t*-test, *P*-value = 0.99; MonoPrep only vs. MonoPrep shared, *t*-test, *P*-value = 0.33).

Statistical analyses of protein abundance per biological feature (Table EV9) were performed using Mann–Whitney *U*-test using the R statistical environment. Variances homogeneity was confirmed in each comparison group using SPSS software. Statistical analyses of protein detection per biological feature (Table EV9) were performed using Fisher’s test (alpha = 5%) on GraphPad software. Statistical analysis of survival and re-expansion time of cryopreserved samples pre-treated with microsuction and controls was performed using Fisher’s test (alpha = 5%) on GraphPad software.

Logistic regression analysis of GAPDH and ACTA levels, H2A family protein detection, and embryo chromosomal status was performed using the R statistical environment (R Core Team, 2015). Specifically, GAPDH and ACTA levels, and the H2A family protein detection were considered for the prediction of embryo’s chromosomal status. These parameters were used in the analysis, as they were the only variables measured in all the tested samples. We used logistic regression to compute the probability of the chromosomal status being euploid, given the values of the three predictors in each sample. A cutoff of 50% for this probability was used for classifying the chromosomal status of a sample.

The classifier was then evaluated using cross-validation with three folds and 20 repetitions as implemented in the “crossval*”* R package (crossval: Generic Functions for Cross Validation. R package version 1.0.2., http://cran.r-project.org/package=crossval). Briefly, the data was split into three parts, two of which were used to train the classifier and one was used to test the classifier (i.e., predict embryo’s chromosomal status). These predictions were then compared to the true chromosomal status. This test was repeated 20 times. Within each cross-validation loop, a variable selection using the three potential predictors was performed using best subset regression with the AIC criterion as implemented in the “bestglm” R package (bestglm: Best Subset GLM. R package version 0.34., http://cran.r-project.org/package=bestglm). This analysis showed that ACTA was hardly ever part of the best model (only in 7% of the cases), while GAPDH levels and H2A protein family detection were important predictors, being part of the best model in 92% and 97% of the cross-validation runs, respectively.

Note that due to the low sample size, each of the three folds contained only 4 or 5 samples. The classifier was always able to predict the chromosomal status; hence, an accuracy of 100% was obtained. However, it is important to keep in mind that the prior selection of only three features could possibly constitute an implicit *a priori* variable selection that could lead to biased cross-validation-based accuracy estimates (Ambroise & McLachlan, 2002). Unfortunately, since we could only measure three potential predictors in all of the samples, it is not possible to elucidate this problem with the current dataset.

## Supporting Information

**Expanded View Figures PDF**

**Table EV1**

**Table EV2**

**Table EV3**

**Table EV4**

**Table EV5**

**Table EV6**

**Table EV7**

**Table EV8**

**Table EV9**

**Movie EV1**

**Movie EV2**

**Review Process File**
