# Supplementary material for: Characterization and quantification of proteins secreted by single human embryos prior to implantation
Source: EMBO Mol Med. 2015 Oct 16;7(11):1465–79. doi: 10.15252/emmm.201505344 (PMC4644378; doi:10.15252/emmm.201505344)
Supplement: Supplementary file 1 [file emmm0007-1465-sd1.pdf]

Expanded View Figures

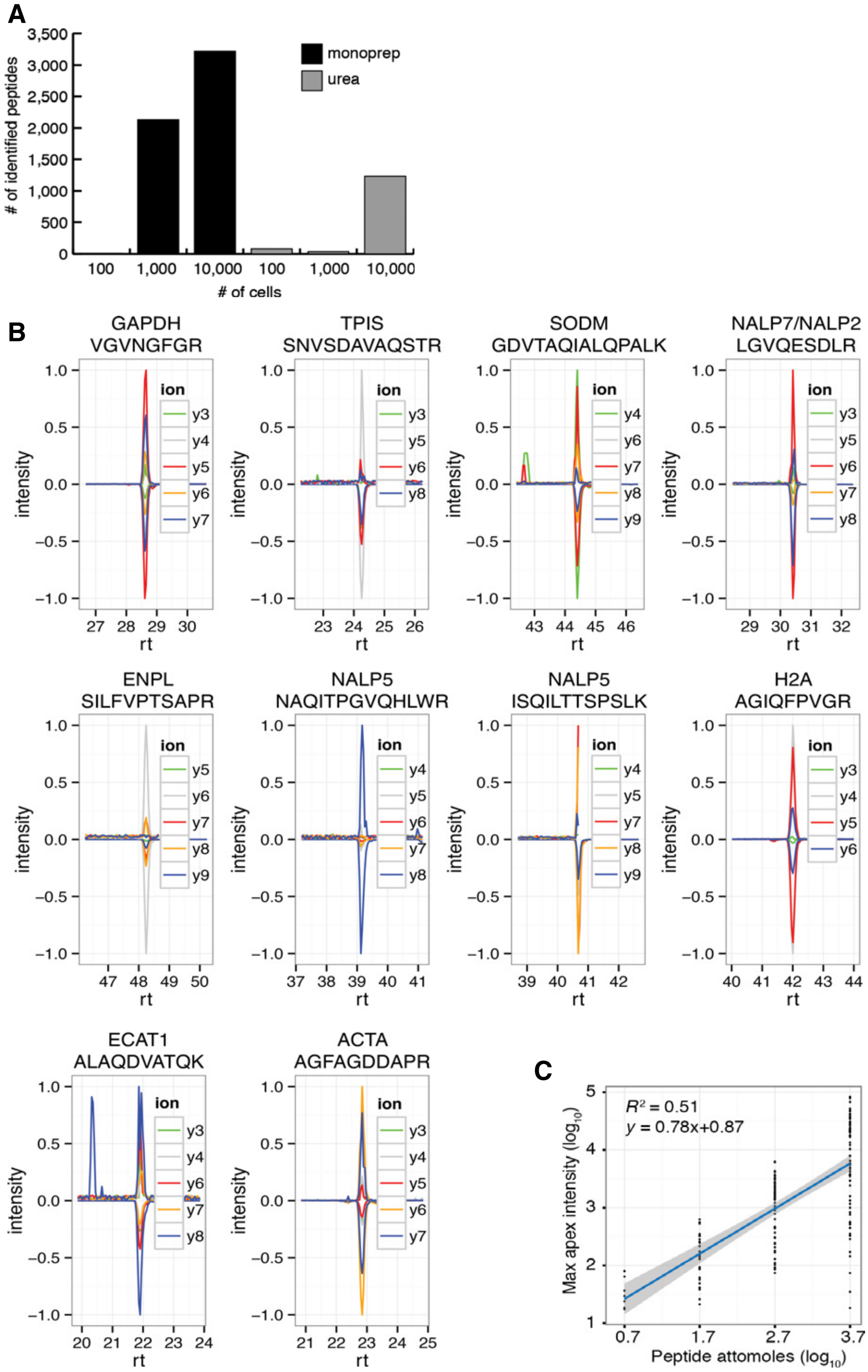

Figure EV1. Comparison of proteomic workflows for blastocoel analysis and development of targeted proteomic assays for single embryo protein quantification.

A Number of peptides identified using different sample preparation methods (black: solvent-based, MonoPrep; gray: urea-based, standard) on cytosolic lysates of HeLa cells at different concentrations (100, 1,000 and 10,000 cells corresponding to an estimated protein amount of 8, 80 and 800 ng, respectively).

B Validation of ten SRM assays on samples of five blastocoels. Batched samples obtained from 5 blastocoels were spiked with isotopically labeled versions of the target peptides. SRM assays were manually validated on the basis of the co-elution and relative intensities of at least 4 transitions per assay. Transition traces are displayed using their relative intensities (with the intensity of the highest transition per assay being set to 1). Positive values are used for endogenous peptides, while negative values are used for reference (synthetic) peptides. Horizontal axis displays peptide retention time (rt) in minutes.

C Intensity calibration curve derived from 76 absolutely quantified (AQUA) peptides measured at different concentrations. The linear regression between log_{10}-transformed maximum apex intensity (derived from the maximum of the most intense transition trace) and log_{10}-transformed attomoles of peptide injected was used to transform SRM-derived peptide intensities for blastocoel proteins into absolute abundances.

© 2015 The Authors

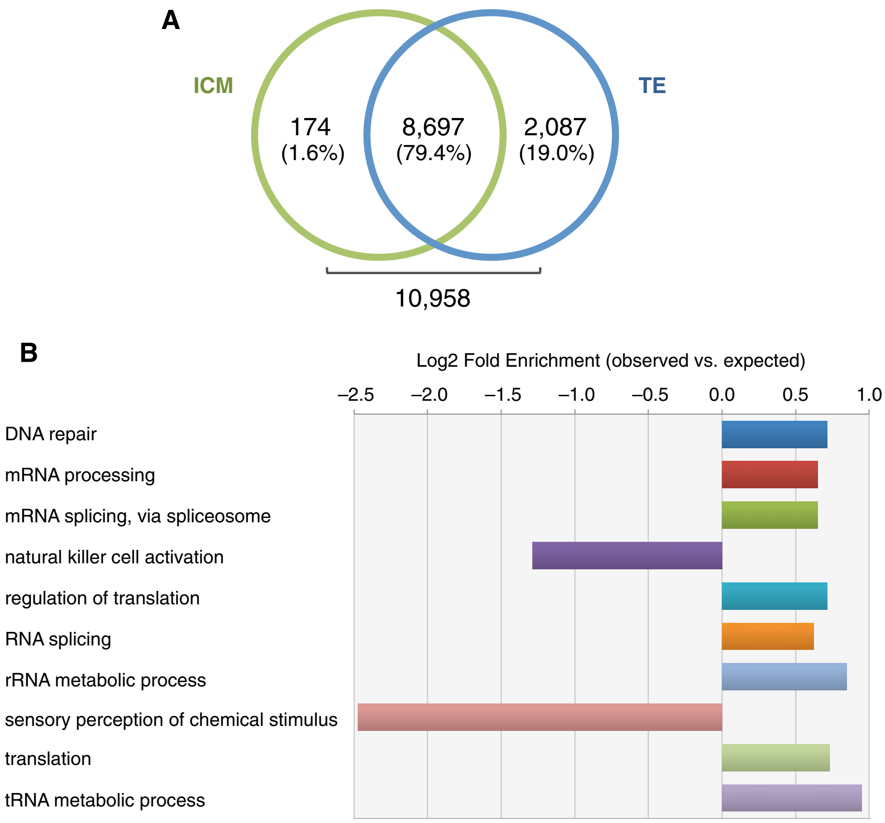

Figure EV2. Transcript detection in individual embryonic tissues and graphical illustration of Panther statistical gene over-representation analysis in human blastocysts samples (Thomas *et al*, 2006; Mi *et al*, 2013).

A Actively transcribed genes in ICM (green) and TE (blue) cells. No transcript showed significant differential expression.

B Blastocyst gene expression investigated using Panther database statistical overrepresentation test comparing global blastocyst transcript list to the default human whole-genome list, which included all genes present in the Panther database. Only biological processes of actively transcribed genes in the human blastocyst with fold enrichment < 0.5 and > 1.5 and *P*-value < 0.01 are shown. Bar lengths are displayed as Log_2 of the ratio between observed and expected number of genes for each category. Raw data and *P*-values for each category are shown in Table EV3.

© 2015 The Authors

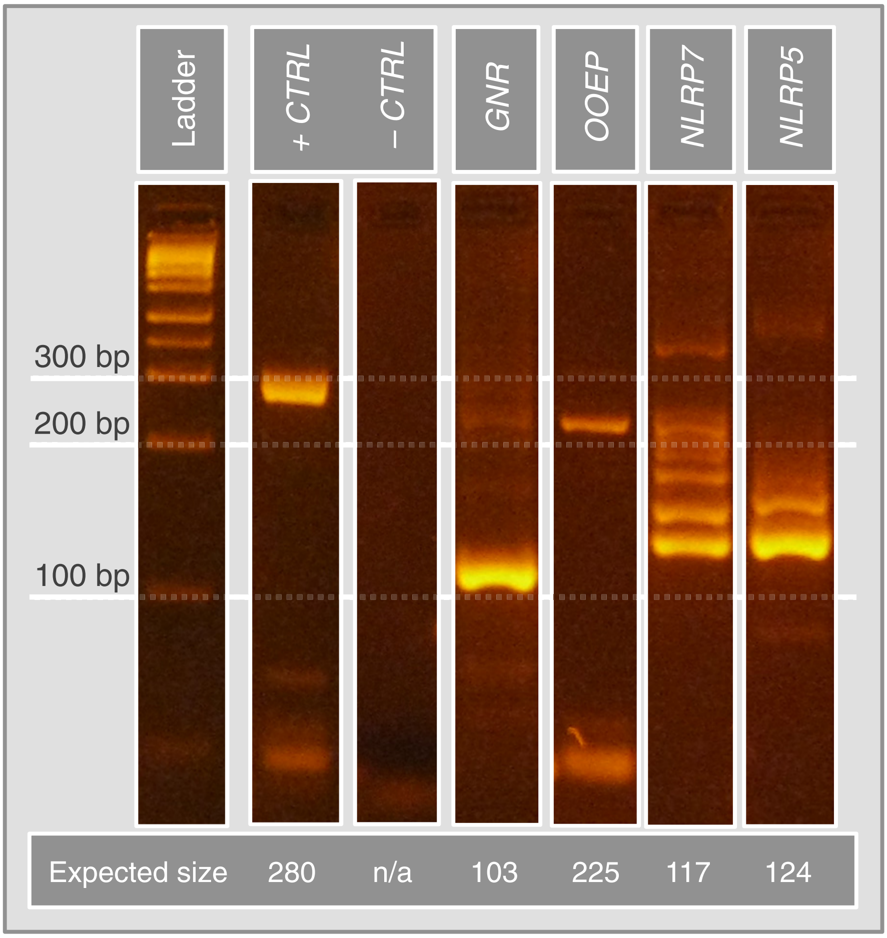

Figure EV3. Validation of embryonic origin of selected MS-identified proteins using PCR.
Total mRNA was isolated from 9 whole blastocysts**.** Four target transcripts (granulins, GRN; oocyte-expressed protein, OOEP; NACHT, LRR, and PYD domains-containing proteins 5, NLRP 5; and NACHT, LRR, and PYD domains-containing proteins 7, NLRP 7) were amplified using nested PCR and custom-built primer sets. Expected bands were excised from the gel and subjected to Sanger sequencing to confirm the presence of the correct transcripts.

 © 2015 The Authors
