# Supplementary material for: Characterization and quantification of proteins secreted by single human embryos prior to implantation
Source: EMBO Mol Med. 2015 Oct 16;7(11):1465–79. doi: 10.15252/emmm.201505344 (PMC4644378; doi:10.15252/emmm.201505344)
Supplement: Supplementary file 13 [file emmm0007-1465-sd13.pdf]

EMBO Molecular Medicine

Characterization and quantification of proteins secreted by single human embryos prior to implantation

Maurizio Poli, Alessandro Ori, Tim Child, Souraya Jaroudi, Katharina Spath, Martin Beck and Dagan Wells

Corresponding author: Dagan Wells, University of Oxford

Review timeline:

Submission date:	15 April 2015
Editorial Decision:	08 May 2015
Revision received:	29 July 2015
Editorial Decision:	20 August 2015
Revision received:	03 September 2015
Accepted:	15 September 2015

Transaction Report:

(Note: With the exception of the correction of typographical or spelling errors that could be a source of ambiguity, letters and reports are not edited. The original formatting of letters and referee reports may not be reflected in this compilation.)

Editor: Céline Carret

1st Editorial Decision 08 May 2015

Thank you for the submission of your manuscript to EMBO Molecular Medicine. We have now heard back from the three referees whom we asked to evaluate your manuscript.

You will see that all three referees find the data of great clinical interest however they all have concerns that have to be addressed in the next version of your article. As you will see below, the main common issue is about the preliminary feel of the findings. However, to paraphrase referee 3, should you be able to "significantly strengthen at least one of these aspects (technical achievement or biological results)", we would be happy to consider a revision. Please carefully provide additional details and clarifications when recommended. Referees also suggested further statistical analyses to increase the conclusiveness of the findings and we would really insist on that. We would like to ask you to focus on addressing the concerns of referees #1 and #3. In addition, if you have data on hand addressing the concerns of referee #2, point 2, we would encourage you to include these in the manuscript, but we would not, ask you to comply with any further-reaching requests.

Given these evaluations, I would like to give you the opportunity to revise your manuscript, with the understanding that the referee concerns must be fully addressed and that acceptance of the manuscript would entail a second round of review. Please note that it is EMBO Molecular Medicine policy to allow only a single round of revision and that, as acceptance or rejection of the manuscript will depend on another round of review, your responses should be as complete as possible.

EMBO Molecular Medicine has a "scooping protection" policy, whereby similar findings that are published by others during review or revision are not a criterion for rejection. Should you decide to submit a revised version, I do ask that you get in touch after three months if you have not completed

© EMBO

it, to update us on the status.

Please also contact us as soon as possible if similar work is published elsewhere. If other work is published we may not be able to extend the revision period beyond three months.

I look forward to receiving your revised manuscript.

***** Reviewer's comments *****

Referee #1 (Comments on Novelty/Model System):

The study is performed by an outstanding team of investigators, it is well written, uses state of the art techniques and it is technically sound. The use of human embryos makes it highly valuable and worth of publication. The use of proteins secreted from the embryo to select viable vs, non-viable embryos has high clinical value.

I am not an expert in proteomics, so a reviewer with expertize with the technique should be contacted to confirm the validity of the technical aspect of the technique. The technique appears appropriate to me

Referee #1 (Remarks):

Poli et al performed an extensive proteomic characterization of the blastocoel fluid (by "blastocentesis") in human preimplantation embryos. Embryos (both frozen and fresh) were donated to research. In particular the authors
1. Collected blastocoel fluid from blastocysts (4-6nL)
2. Performed proteomics analysis of blastocoel fluid using 2 techniques
a. Urea prep: this approach was only feasible for pooled samples (two sets of n=20 samples)
b. Mono prep, that could be optimized for individual samples (two sets of n=20 samples)
3. Performed Single proteomic analysis of 9 target proteins in single embryos
4. Performed microarray analysis and validated 4 genes (GRA, OOEP, NLRP5 and NLRP7) by PCR and sequencing using mRNA from 9 pooled blastocysts
5. Performed cytogenetic analysis of 14 fully expanded blastocysts previously subjected to blastocentesis using a-CGH.
The authors found a total 288 proteins
1. 169 proteins were presents in the urea prep. 76 out of 169 (45.0%) proteins identified with Urea-based preparations were actively transcribed in the embryo,
2. 150 from the mono prep; 121 out of 150 (80.7%) were confirmed by microarrays
3. Further they correlated GAPDH and H2A protein levels to ploidy status in 14 blasts and found that absence of H2A and abundance of GAPDH had a 100% accuracy to identify euploid embryos.

The study is performed by an outstanding team of investigators, it is well written, uses state of the art techniques and it is technically sound. The use of human embryos makes it highly valuable and worth of publication. There are few major critiques
1. It is unclear how many embryos were used for all the extensive molecular and viability characterizations of the embryos (for example: page 8: 50 blasts underwent micro-suction and cryopreservation: were any of these blasts used for molecular analysis?). A table summarizing how many embryos were used for each analysis and if embryos were used for multiple testing should be provided
2. The only weak part of the paper is the correlation of the GAPDH and H2A levels to ploidy status. First, the sample size is extremely low. Second it is unclear why only GAPDH and H2A were chosen among the hundreds of proteins available. Indeed an unbiased and unsupervised statistical correlation of all the proteins measured and the ploidy status should be performed. As presented, the results are misleading and should not be included in the paper unless more samples are tested and a global protein-to-ploidy correlation is performed.
3. Microarray data: There is no description of how many embryos were used for microarray analysis

(single embryo? Pooled embryos?). How many replicates were performed?
a. Were all the embryos derived from fresh culture or some from frozen cycles? If both, the gene expression results could be different because of the freezing and thawing process. In this case, the data should be re-presented separating the two groups of embryos.
b. Overall a more extensive analysis of data is needed. These are very valuable results that deserve additional data mining.
There are other minor critiques
4. The authors, (page 6) states that 177 embryo derived proteins were identified. However it is unclear how these numbers come about given that 150 and 169 proteins were identified with the 2 different methods. A Venn diagram showing how many proteins were found in both mono prep and urea prep is needed to clarify this.
5. Bottom of page 6: ...173 proteins have known functionality... this phrase is unclear. Please rewrite.
6. Figure1a: add the n number of embryo tested
7. Table 1: how and what criteria did the authors use to present only a subgroup of proteins in table 1 out of the 100s available?
8. Page 14: it is unclear if the embryo that score as "C" are defined as poor morphology. Reference 36 is not correct.
9. In Figure S1: the graph could be larger for better visualizations. Also specification of what the abbreviations stands for (rt) should be provided. Please explain why some graphs have 2 peaks (e.g. ECAT1 and SODM)
10. Supplemental data: tables on page 5, 16, 23 and 29 are not labelled and so it is difficult to identify Supplemental tables. For example, Table S1 could not be found
11. Some of the tables have redundant material: for example on supplemental table page 5: there are columns with the statement "reviewed" or "homo sapiens": this should be removed. Overall the supplemental tables should be made clearer for the reader, removing un-needed information

Referee #2 (Comments on Novelty/Model System):

This very accessible manuscript describes mass spec procedures to obtain non-destructive proteomic profiles from blastocoel fluid with the goal of selecting the best human embryo(s) for transfer in assisted reproductive technologies. Although promising, publication seems premature as no criteria (protein profile) for selecting superior blastocysts was determined. The 288 identified proteins are a hodge-podge of cytoplasmic, nuclear and secreted proteins (76 overlapped with an earlier study - ref #27) and it is not clear what protein profile would predict better developmental competence or be associated with chromosomal abnormalities.

Referee #2 (Remarks):

The tight junctions of the outer trophectoderm of the mammalian blastocyst permit formation of a central blastocoel by facilitated water passage through aquaporins in response to a sodium gradient produced by Na+/K+ ATPases. If the fluid is mechanically removed, it re-accumulates and embryos can implant and gastrulate. No mechanistic or correlative connection has been established between the composition of the blastocoel fluid and success in development. Previous studies have reported on the protein composition of the blastocoel in humans and cows (Stem Cells Dev 22:1126, 2013; Syst Biol Reprod Med 60:127, 2014) and the authors now add to the human catalogue in an eminently readable and technologically sophisticated manuscript.

Comments that the authors may wish to consider:
1. It is not clear that proteins present in the blastocoel (presumably secreted from embryonic cells) are a better source of predictive markers than the culture fluid surrounding 2- or 4- cell embryos. Use of the culture media would avoid any perturbation that might result from manipulations with the aspiration needle.

2. Given the limited amount of material available from human embryo, the use of a model system (mouse) could provide greater depth to their proteomic screen to identify predictive markers for

successful ART. In addition, the conservation of proteins in mouse and human blastocoel fluid would suggest greater importance than those not conserved.

3. The abundance of keratin in the mass spec results raises the possibility of contamination during sample preparation. Is a similar abundance of mouse keratin observed after aspiration of mouse blastocoel fluid?

4. Although the manuscript is very well written, it would benefit from copy editing to ensure compliance with EMBO style of capitalization.

Referee #3 (Comments on Novelty/Model System):

Please see my comments to the authors for details.

Referee #3 (Remarks):

In this manuscript, Poli and colleagues demonstrate that the quantification of proteins in human embryo blastocoels is possible and potentially useful as a screening tool for preimplantation genetic screening (PGS) after in vitro fertilization (IVF). Specifically, they perform blastocentesis, the extraction of few nanoliters of blascocoel fluid from single blastocysts, and characterize the proteome using shotgun proteomics (they use different protocols for single and multiple samples). They validate the identified proteins using whole embryo microarrays to filter out possible contaminants and then select a set of 10 peptides corresponding to 9 protein groups to perform targeted proteomics on each of 21 single blastocoels. Using array-CGH, they perform cytogenic analyses on 14 whole blastocysts, which have undergone blastocentesis and quantitative proteomics, and find that presence of H2A and abundance of GAPDH can predict aneuploidy with 100% accuracy. Finally, they show that blastocentesis has little effect on embryo viability as judged by immediate blastocoel re-expansion, and a positive effect after cryopreservation when compared to controls that have not undergone blastocentesis.

In its current form, the work aims to be a hybrid between technical achievement and biological results, but it struggles to fulfill both aspects. As a general recommendation therefore, I believe that the authors would do well by significantly strengthen at least one of these aspects.

For example, on the technical side, the authors attempted to tackle the experimentally very challenging problem of quantifying proteins via 10 representative peptides from an extremely low volume/amount of starting material, which is very interesting. Unfortunately, they are not able to comprehensively detect and quantify these peptides in all of the samples using SRM, which raises doubts about the sensitivity of the method. In addition, only one proteotypic peptide is used per protein in their SRM assays, which seems substandard given the well-accepted notion that at least 2, ideally 3 or 4 peptides should be monitored to control for intrinsic assay variability. In this regard, while conceptually definitely interesting, it may be a bit premature (unless the authors can convince me otherwise) to think of their procedure as an effective screening tool since already established genetic techniques are simpler and currently more informative. For instance, a recent study by Gianaroli et al. has shown, also using blastocentesis, that DNA is present in blastocyst fluids and could be efficiently used to detect aneuploidy. This DNA could also be used to detect other genetic disorders, which has immediate clinical implications. I think the authors should discuss and cite the Gianaroli paper since it is highly relevant.

On the biological side, I think that the authors should capitalize more on the characterization of the blastocoel proteome, which is a first, and the relationship between protein levels and aneuploidy, rather than on the implications for screening.

Comments and questions:
1- In page 5, some of the identified proteins were not validated by microarrays. The authors assume

that these proteins are contaminants. While this could be the case, the authors do not rule out the possibility of the persistence of proteins of maternal origin. Could the authors discuss how likely this is the case? It would also be interesting to see if any of those proteins is a good predictor of aneuploidy, and thus implicate maternal contributions in the success of IVF.
2- On similar lines, since it has been shown that poor quality sperm could affect preimplantation embryo development (Janny and Menezo, 2005), I wonder whether data about sperm quality is available (sperm count, motility, and morphology), and whether this correlates with aneuploidy or any of the measured protein levels. This could be included in Table S7 (which, by the way, is not referenced in the manuscript). I realize that the sample size is inadequate for statistical analyses, but I think that this information, if available, is interesting to be reported.
3- There is little overlap between the Urea-based preparation and Monoprep samples in terms of identified proteins (I had to calculate it, and it is 31 if I understood well). How many of those were validated by microarrays? Are they more abundant than others? Are they more likely to be contaminants? Some simple statistics could be performed to answer these questions.
4- The authors report the intensities of protein targets measured in single blastocoels in Table S6. With the exception of GAPDH, and as already mentioned earlier, none of the proteins is quantified in all samples. There are actually more missing points than values. The authors do not discuss whether this is due to technical limitations, to fluctuations in the protein levels between different embryos (where in some embryos they are below the detection limits), or to the absence of the protein in some samples. I guess from the trend in figure 3a, one could argue that proteins with missing values are of lower abundance, and therefore technical limitations are to blame.
5- Typically, it is good practice to choose at least two proteotypic peptides per protein or protein group to check for the stability of the SRM assay. One would like to see that the peptides belonging to the same protein correlate with each other. It is not clear to me why the authors have not done this and they should do so if they want to increase the robustness of their results.
6- I do not understand how the authors arrived to their final logistic regression model using the presence of H2A and abundance of GAPDH as predictors. How does each predictor perform alone? And how about other combinations? How do the different protein levels correlate to each other, and do aneuploid embryos have a different "signature" of those 9 proteins? I am not sure that this is possible given the missingness in the SRM data.
7- If the results of the authors have true biological meaning, then one would expect other histones to also correlate with aneuploidy (thus in similar fashion as H2A). Given that histone H4 was one of the most abundant proteins identified in the monoprep procedure, it should be relatively straightforward to also examine the correlation between H4 abundance and aneuploidy. It is unclear why the authors chose not to do this and why they selected histone H2A and not H4 for the SRM measurements?
8- The authors do not discuss how the technique could be possibly improved to increase sensitivity.

Minor comments:

The authors do not refer to figure 3b until the discussion, but it is covered earlier in the text (specifically on p8)
In p7, authors refer to Glyceraldehyde 3-phosphate dehydrogenase as G3P, but in other places as GAPDH.
In the materials and methods, the statistical analysis section has very little information about the logistic regression model used and the cross validation.

References:

Gianaroli et al. Blastocentesis: a source of DNA for preimplantation genetic testing. Results from a pilot study. Fertility and Sterility, 102(6): 1692-1699. (2014)
Janny L. and Menezo Y.J.R. Evidence for a strong paternal effect on human preimplantation embryo development and blastocyst formation.

1st Revision - authors' response 29 July 2015

Response to Reviewers' comments:

We would like to thank all the Reviewers for their constructive comments and their feedback on how to improve our manuscript.
One of the main concerns that emerged from the referees' comments is the necessity to address some of the technical aspects, especially for the statistical analysis performed on the data obtained from single embryos. We have now reviewed and re-analysed all the data generated with the aim of investigating any relationship between the proteins measured and the biological features of the embryo. We now provide detailed description of all the comparisons we performed between protein abundance levels and aspects of embryo biology (**Table EV2**) and justify the selection of GAPDH levels and histone H2A detection for the logistic regression analysis.
In this regard, we would like to point out that due to the extremely small volumes of the samples deriving from single embryos it was only possible to reliably quantify the 3 most abundant proteins (GAPDH, H2A and ACTA) across the majority of the analyzed embryos. In addition to these, we were able to quantify in single blastocoels embryo-specific proteins such as multiple components of the SubCortical Maternal Complex (SCMC) and secreted factors such as Granulins and Serpin family proteins. Unfortunately, the limit of detection of our approach based on targeted proteomics (~5 attomoles) did not allow quantification of these proteins across a sufficient number of single embryo blastocoels to provide a comprehensive proteomic profile of this challenging specimen. However, GAPDH level and H2A detection tended to correlate with ploidy status when analyzed individually (**Table EV2**). Reassuringly, when those two signals were combined in a logistic regression model they proved to correlate and reinforce each other (**Figure 3C**) achieving 100% accuracy in our, admittedly limited, cohort of samples.
We clearly state that these findings should be treated with caution due to the small sample size. We additionally state that additional work is necessary in order to establish whether GADPH and H2A protein level in the blastocoel can be considered as biomarker for the ploidy status of the embryo (line 365, page 14). However, we believe that our work opens up the exciting perspective of measuring single-embryo-derived proteins that might be indicative of embryo status, providing a valuable foundation for future work. We are convinced that the steady improvement of the sensitivity of proteomic technologies will enable in the near future this kind of analysis to become more comprehensive and might offer a more direct determination of the functional status of an embryo than current techniques.

Unfortunately, we are not in the position to generate additional targeted proteomic data on blastosol samples in a reasonable time frame. We would like to stress that the embryos used in this study derive from IVF treatments where patients had previously consented to donate surplus embryos to this specific experimental project. Therefore these samples represent extremely precious material that cannot be easily obtained. Currently, supernumerary embryos are of limited availability within our department (and indeed they are a rare resource worldwide) and need to be shared with other ongoing projects.

Nonetheless, we provide full details on the protocols used to obtain these preliminary

results. We hope that this will stimulate further research and that the information will be used by other groups to provide support to our initial findings. We believe that independent validation of the methodology we describe would be even more valuable than analysis of more samples in our laboratories. We have provided full disclosure of our protocols to assist any group wishing to replicate and expand upon this work.

Despite the inability to increase the sample size further, we believe that we have successfully addressed all other points raised by the Reviewers and we hope these improvements are well received.

Our detailed point-by-point response follows below.

REFEREE 1

1. It is unclear how many embryos were used for all the extensive molecular and viability characterizations of the embryos (for example: page 8: 50 blasts underwent micro-suction and cryopreservation: were any of these blasts used for molecular analysis?). A table summarizing how many embryos were used for each analysis and if embryos were used for multiple testing should be provided

We agree with this comment and opted for a graphical visualization of embryo usage in the experiments carried out in the study. The figure showed below is included in the revised **Figure 1**.

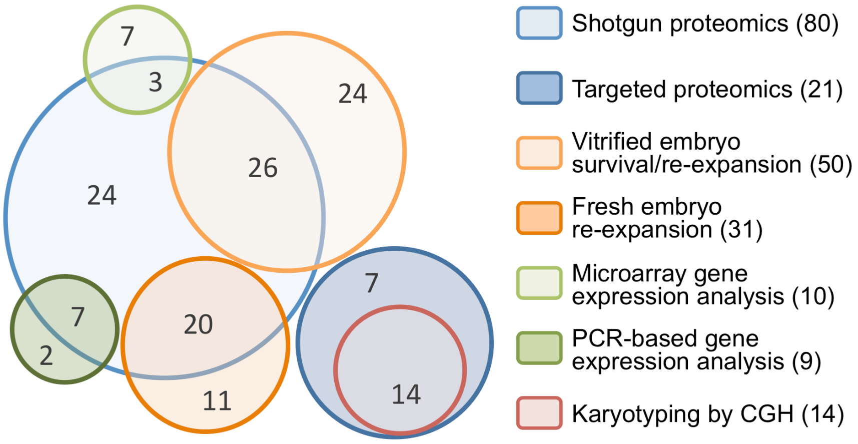

Figure 1C

"Embryo usage map – Each circle represents the embryo samples used in specific experimental set (Blue, proteomics; Orange, Embryology; Green, Gene expression and Red, Cytogenetics). Total number per technique is shown in brackets in the legend. In the circles, numbers correspond to the number of samples used for each experiment."

2. The only weak part of the paper is the correlation of the GAPDH and H2A levels to ploidy status. First, the sample size is extremely low. Second it is unclear why only GAPDH and H2A were chosen among the hundreds of proteins available. Indeed an unbiased and unsupervised statistical correlation of all the proteins measured and the ploidy status should be performed. As presented, the results are misleading and should not be included in the paper unless more samples are tested and a global protein-to-ploidy correlation is performed.

These are valid points raised by the Referee.
Firstly, we agree that a larger sample size for correlating protein abundance with ploidy status would be extremely beneficial. However, as explained above, presently we are not in a position to be able to process more individual embryonic samples for protein and genetic analysis.
Secondly, we agree that the reason why we focused on GAPDH and H2A as parameters for the logistic regression analysis was not clear in the text. In order to clarify this point, we

introduced a table showing all data used for statistical testing across all comparison groups. As shown in **Table EV2**, GAPDH and H2A were the two proteins that when considered individually produced the lowest P-values following Mann-Whitney U test and Fisher's test, (P-value 0.029 and 0.056 respectively, see table below). Also, both analyses were generated in the karyotype comparison groups. For this reason, a logistic regression analysis was performed in the original manuscript using only these two parameters as predictors of aneuploidy. In the revised manuscript, we have included also actin family as third predictor, being the only other protein that was quantified across the majority of single blastosols analysed. Within the logistic regression analysis, we allowed a variable selection between the three predictors in each cross validation step. While GAPDH levels and H2A protein family detection were included 92% and 97% of the time respectively, the ACTA family was only included in 7% of the cross validation loops. This confirms that in our cohort of samples the levels of GAPDH and H2A protein family have superior discriminative power of the plody status of the embryos.
We believe that including the table summarizing the relationship between all proteins investigated and embryonic features provides additional transparency on the methodology used in the statistical analysis section (**Table EV2**). We also introduced an additional paragraph in the "**Cytogenetic analysis of whole blastocysts"** section (line 230, page 9)**,** where we clarify the decision to use GAPDH and H2A in the logistic regression analysis.

As shown in **Appendix Table S7**, the data points collected for each individual sample are not sufficient to generate a blastosol protein profile containing more that 3 proteins (GAPDH and ACTA levels, H2A detection). Although desirable, unfortunately it is not possible with the present data to generate a complete Protein-to-ploidy correlation as suggested by the Reviewer. Technical limitations preclude targeted analysis of such a large number of proteins in individual blastosol samples and consequently such analysis would require the collection of multiple blastosols with many subsequent rounds of targeted proteomics analysis, each assessing small numbers of proteins. While this would eventually allow us to build up a comprehensive picture of the relative quantities of proteins in individual samples a large number of very hard to obtain embryos would be required (consider that triplicate analysis is required expanding the number of samples needed even further). Additionally, each embryo would need to be subjected cytogenetic analysis. This could ultimately mean array-CGH analysis of hundreds of samples, which would be prohibitively expensive. We believe this kind of an analysis would be extremely valuable, however it would go beyond the scope of the current work. Nonetheless, we appreciate the validity of the Reviewer's comment and we hope to continue investigation into the significance of the relationship of protein profile and ploidy status in future studies to the maximum extent that sample availability and funds will allow.

Protein	Median±MAD, n	Median±MAD, n	P-value
KARYOTYPE	Euploid	Aneuploid	
GAPDH	3.294 ± 0.168, n=9	3.675 ± 0.285, n=5	0.029
ACTA	2.639 ± 0.511, n=9	2.763 ± 0.426, n=5	0.317
SEX	Female	Male	
GAPDH	3.483 ± 0.261, n=7	3.2375 ± 0.706, n=6	0.295
ACTA	2.639 ± 0.458, n=7	2.829 ± 0.632, n=6	1
MORPHOLOGY	Good	Poor	
GAPDH	3.300 ± 0.023, n=4	3.419 ± 0.449, n=10	0.539
ACTA	2.509 ± 0.327, n=4	2.829 ± 0.408, n=10	0.436
PT AGE	<35	≥35	
GAPDH	3.307 ± 0.546, n=7	3.483 ± 0.280, n=7	0.318
ACTA	2.763 ± 0.377, n=7	2.639 ± 0.609, n=7	0.443
SEMEN	Normal (>15M, >40%)	Poor (<15M,<40%)	
GAPDH	3.483 ± 0.424, n=7	3.336 ± 0.089, n=7	0.805
ACTA	2.763 ± 0.510, n=7	2.639 ± 0.385, n=7	0.798
INSEMINATION	IVF	ICSI	
GAPDH	3.389 ± 0.436, n=6	3.346 ± 0.234, n=8	0.95
ACTA	2.734 ± 0.632, n=6	2.701 ± 0.359, n=8	0.846

	H2A family detected	H2A family not detected	P-value
Euploid	3	6	0.056
Aneuploid	5	0	
Female	3	4	0.59
Male	4	2	
Good Morph	1	3	0.24
Poor Morph	7	3	
Pt Age <35	3	4	0.59
Pt Age >35	5	2	
IVF	4	2	0.63
ICSI	4	4	
Normal Semen	5	2	0.59
Poor Semen	3	4	

Table EV2

3. Microarray data: There is no description of how many embryos were used for microarray analysis (single embryo? Pooled embryos?). How many replicates were performed?

a. Were all the embryos derived from fresh culture or some from frozen cycles? If both, the gene expression results could be different because of the freezing and thawing process. In this case, the data should be re-presented separating the two groups of embryos.

b. Overall a more extensive analysis of data is needed. These are very valuable results that deserve additional data mining.

The Reviewer made a well founded point. We agree that we didn't include sufficient details on the samples used for microarray analysis and we have introduced a paragraph explaining sample population composition and characteristics. All tested samples were considered as fresh blastocysts. Seven of these derived from embryos frozen on Day-3 and then thawed and cultured to Day-5, however we do not feel appropriate to define these samples as frozen blastocysts. A paragraph addressing Reviewer's comment can now be found in the **"Gene expression analysis - Microarrays"** section in Materials and Methods (line 543, page 20) stating:

"Data were obtained from a total of ten embryos. For each embryo, Inner Cell Mass and Trophectoderm were separated and distributed into three paired replicates (three ICM and

three TE). Two pairs were composed of pooled cellular material from three and one pair from four blastocysts. Each replicate was analysed separately and the list of active genes were combined. Embryos used in these experiments were Day 5/6 embryos deriving from fresh treatment cycles or embryos that were thawed at Day 3 of development and then cultured to blastocyst stage"

We strongly agree with the Reviewer that the data generated on blastocyst gene expression are very valuable, however we would respectfully point out that the purpose of the human blastocyst gene expression analysis in this paper is to provide an insight into whether the proteins detected using shotgun mass spectrometry are of embryonic origin. Also, this data helped to reveal the higher specificity of the MonoPrep protocol. Although we feel that a comprehensive analysis of the transcriptomic data probably isn't appropriate for the current paper, we have added some additional information, which we hope might be of interest. This takes the form of a gene overrepresentation analysis performed using the Panther gene expression analysis tool. Results are now reported on **Appendix Table S3**, mentioned in section **Gene expression analysis in embryos** (line 129, page 5) and shown in **Figure EV2**.
It is our intention to carry out a more in depth analysis of the data collected about human blastocyst transcriptome, corroborated by other findings, in a separate publication, allowing a greater focus on gene expression than could be achieved in the current paper. We also deposited all of the raw microarray data in the Gene Expression Omnibus (GEO) repository (Accession code GSE71455).

PANTHER GO-Slim Biological Process	Homo sapiens REFLIST	Transcripts identified	Transcripts expected	Transcript Over/Under representation	Transcripts Fold enrichment	Bonferroni adjusted P-value	Log2 Fold enrichment
DNA repair	172	137	83.75	+	1.64	1.14E-05	0.71
mRNA processing	274	210	133.42	+	1.57	9.39E-08	0.65
mRNA splicing, via spliceosome	183	140	89.11	+	1.57	7.48E-05	0.65
natural killer cell activation	99	20	48.21	-	0.41	7.77E-04	-1.29
regulation of translation	148	118	72.07	+	1.64	8.68E-05	0.71
RNA splicing	135	101	65.74	+	1.54	6.74E-03	0.62
rRNA metabolic process	115	101	56	+	1.8	8.16E-06	0.85
sensory perception of chemical stimulus	133	12	64.76	-	0.18	2.00E-13	-2.47
translation	435	352	211.82	+	1.66	7.40E-17	0.73
tRNA metabolic process	82	77	39.93	+	1.93	2.61E-05	0.95

Appendix Table S3.

"These data were analysed using Panther Classification System gene over-representation analysis tool, based on GO-slim biological process annotation. As shown on Table S3, the majority of the biological activities showing 1.5 fold gene enrichment compared to a default human cell transcriptome gene list involve both transcription and translation processes. This increased number of active genes involved in tRNA and rRNA metabolic processes, combined with high transcription of genes required for regulation of mRNA maturation and translation, confirm an extremely active biogenesis activity in the developing blastocyst. Interestingly, genes involved in local immunological modulation (NK-cell activation and sensory perception of chemical stimulus) appear to be down regulated, potentially to minimize host's inflammatory reaction to embryonic presence."

There are other minor critiques

3. The authors, (page 6) states that 177 embryo derived proteins were identified.

However it is unclear how these numbers come about given that 150 and 169 proteins were identified with the 2 different methods. A Venn diagram showing how many proteins were found in both mono prep and urea prep is needed to clarify this.

On Page 5 we explain that only some of the genes corresponding to proteins identified with shotgun MS were definitely shown to be expressed by the embryo, as the corresponding mRNA was detected using microarrays (see also **Figure 2A**). We reanalyzed the data in order to provide the information requested regarding the proteins identified with both procedures. We found that there was support (from gene expression studies) for the active production of 80/169 proteins detected using the Urea method and 123/150 proteins from MonoPrep, for a total of 203. This differs slightly from the figures previously given, a discrepancy now corrected throughout the text. 21 of these proteins were identified with both techniques, giving a final number of 182 actively expressed proteins detected with the two methods combined. We have included a figure (**Figure 2A**) showing how many proteins were identified with both techniques.

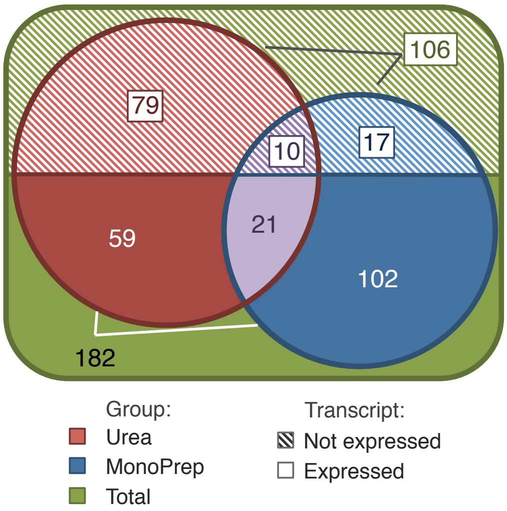

Figure 2A

4. Bottom of page 6: ...173 proteins have known functionality... this phrase is unclear. Please rewrite.

On the other hand, 173 proteins have known functionality within the cytoplasm and are related to metabolic processes.

We rephrased the sentence in question (line 193, page 8):

"On the other hand, 173 proteins are annotated as localized in the cytoplasm where they take part in metabolic processes."

5. Figure1a: add the n number of embryo tested

With the introduction of the Venn diagram in **Figure 1**, we believe this point is addressed. (See also reply to Point 1)

6. Table 1: how and what criteria did the authors use to present only a subgroup of proteins in table 1 out of the 100s available?

Since the tables containing all information about proteins identified in the blastocoel was too large to be included in the main text, we assembled **Table 1** to provide some interesting summary information about the most abundant proteins detected. This is provided in order to avoid the reader having to refer to the supplementary data. However, the more comprehensive listing is available to those who are interested.
From the total protein identification catalogue, the subset presented in **Table 1** includes only those proteins identified with a False Discovery Rate lower than 1% that were not present among the proteins identified in blank samples (which were considered to be contaminants). In the "most abundant proteins" section Urea and MonoPrep groups were kept separated to show that a smaller proportion of the proteins detected in MonoPrep samples were attributable to contaminants, demonstrated by the lower ordinal number in the abundance rank column.
Lower boxes for each group give examples of proteins in lower concentrations that have functions related to embryonic development.

To clarify the criteria used to include the proteins in **Table 1**, we introduced a sentence in the figure legend stating:

"Proteins identified in blank samples were considered as common contaminants and removed from the catalogue of identified proteins obtained from blastosol samples. The abundance ranking column refers to the relative protein intensity levels prior to exclusion of common contaminants. The proteins detected using the urea-based method shows less continuous numbering of 'abundance rank' because a relatively large number of high abundance contaminants were detected and excluded."

8. Page 14: it is unclear if the embryo that score as "C" are defined as poor morphology. Reference 36 is not correct.

Reference 36 provides the criteria we used to grade the embryos. The reference does not provide a definition of good or poor quality embryo. Instead, on page 14, in the Materials and Methods section, we describe the method used to score the embryos GOOD or POOR.
To clarify the group assignment of embryos with score C we introduced the following sentence in Material and Methods **Blastocyst morphological assessment** section (line 457, page 17):

"Embryos that showed ICM or TE of C grade were considered GOOD if the other parameter was an A or a B, and POOR if the other parameter was a C, D or E."

Also, we also added as a reference the original book chapter that explains blastocyst assessment criteria by Gardner DK (Reference 40).

Gardner, D. K., & Leese, H. J. (1999). Assessment of embryo metabolism and viability. In Handbook of In Vitro Fertilization 2nd edn (pp. 347–372).

9. In Figure S1: the graph could be larger for better visualizations. Also specification of what the abbreviations stands for (rt) should be provided. Please explain why some graphs have 2 peaks (e.g. ECAT1 and SODM)

We have now revised **Figure EV1** to improve readability. "rt" indicated peptide retention time and it is now explicitly stated in the figure legend. SODM and ECAT1 show additional peaks deriving from interfering signals. It has to be noted that these signal affect only one of the channels (the "light" endogenous peptide in these cases) and derive only from two (y4 and y7 for SODM) and one transition (y8 for ECAT1). For these reasons, these signals do not interfere with the detection and quantification of the correct peak group for the selected peptides that is based on the detection of 5 co-eluting transitions for both the light (endogenous) and heavy channel (spiked-in synthetic peptide).

10. Supplemental data: tables on page 5, 16, 23 and 29 are not labelled and so it is difficult to identify Supplemental tables. For example, Table S1 could not be found

Unfortunately, this was due to some difficulties in the formatting of the material when uploaded on the website. This issue was addressed introducing the reference name directly on the table and not only on the file name. This should improve Supplementary Material identification.

11. Some of the tables have redundant material: for example on supplemental table page 5: there are columns with the statement "reviewed" or "homo sapiens": this should be removed. Overall the supplemental tables should be made clearer for the reader, removing un-needed information

Noted and addressed.

REFEREE 2:

2. Given the limited amount of material available from human embryo, the use of a model system (mouse) could provide greater depth to their proteomic screen to identify predictive markers for successful ART. In addition, the conservation of proteins in mouse and human blastocoel fluid would suggest greater importance than those not conserved.

We agree with the Reviewer's comment and concur that samples from a mice model would be more readily collected. The identification of conserved proteins might well assist in the identification of functional/developmental proteins with a key role in fundamental

processes and for this reason we are considering future work in model organisms. However, most of the abnormal features seen in human embryos, which have a significant impact on their potential to form a viable pregnancy, are only rarely seen in murine embryos (e.g. aneuploidy, fragmentation, cleavage stage arrest). The relative infrequency of these problems has led to some doubts about the suitability of the mouse for the identification of clinically relevant biomarkers applicable to humans. Unfortunately, we do not currently have any data on whether murine and human blastocoels have similar protein contents.

REFEREE 3

In its current form, the work aims to be a hybrid between technical achievement and biological results, but it struggles to fulfill both aspects. As a general recommendation therefore, I believe that the authors would do well by significantly strengthen at least one of these aspects.

For example, on the technical side, the authors attempted to tackle the experimentally very challenging problem of quantifying proteins via 10 representative peptides from an extremely low volume/amount of starting material, which is very interesting. Unfortunately, they are not able to comprehensively detect and quantify these peptides in all of the samples using SRM, which raises doubts about the sensitivity of the method. In addition, only one proteotypic peptide is used per protein in their SRM assays, which seems substandard given the well-accepted notion that at least 2, ideally 3 or 4 peptides should be monitored to control for intrinsic assay variability. In this regard, while conceptually definitely interesting, it may be a bit premature (unless the authors can convince me otherwise) to think of their procedure as an effective screening tool since already established genetic techniques are simpler and currently more informative. For instance, a recent study by Gianaroli
et al. has shown, also using blastocentesis, that DNA is present in blastocyst fluids and could be efficiently used to detect aneuploidy. This DNA could also be used to detect other genetic disorders, which has immediate clinical implications. I think the authors should discuss and cite the Gianaroli paper since it is highly relevant.

We thank the Reviewer for his/her comment on our manuscript. We would like to respectfully point out that we are not claiming that our proteomic approach represents an effective screening tool for embryo assessment yet, and we agree with the Reviewer that there may be more established methodologies to detect embryo aneuploidy, such as cytogenetic analysis of biopsied cells. Nonetheless, we hope to persuade the Reviewer of the importance of our study as proof of principle for protein detection in single blastocoel samples, a technical achievement never reported before. According to our experience in preimplantation genetic assessment, embryonic cytogenetic analysis does not provide 100% assurance that the embryo is able to implant and generate a healthy pregnancy. In fact, approximately one-third of morphologically normal euploid embryos fail to implant. It is therefore a common idea that other processes come into play to define the competence of an embryo to implant. We believe that one of these could be the metabolic and functional state of the embryo. For these reasons we focused on a technique that could reveal some of

these aspects. It is our belief that new methodologies should be applied experimentally with the aim to provide additional diagnostic power to embryo assessment strategies. Due to ethical and legal limitations, we could not transfer the embryos we tested back to the patients that donated them, therefore we used embryo cytogenetics as a reference to identify potentially viable versus non-viable embryos. We hypothesize that implanting and non-implanting embryos may express different amounts of key proteins. The identification of such proteins will be a significant undertaking and is beyond the scope of the current paper. Nonetheless, we hope that this study demonstrates the principle of how such research can be undertaken and lays vital technical groundwork for future studies.

We concur that the paper of Gianaroli and colleagues paper can be seen as relevant to our work and we have introduced a sentence mentioning it in the introduction section (line 79, page 3) and included its reference in the bibliography (Ref 8 to 10).

We agree with the Reviewer that the technique requires refinements and extensive validation before it can be proposed as a diagnostic tool. However, at the time we started this study, targeted proteomics offered the most direct approach to protein identification and quantification. It may also be possible that SRM assays employed to identify selected protein candidates were not the most sensitive or informative and other methodologies should be investigated. We have now discussed this relevant aspect in the discussion section (line 365, page 14). We believe that the continuous improvement of proteomics devices, such as mass spectrometers or electronic ELISA detectors, will enable in the near future protein abundance measurement of more targets, allowing for a more comprehensive proteomic profiling of the blastocoel.

It is our opinion that the future of embryo assessment does not lie in the use of a single methodology but it will require a comprehensive approach, able to assess different features of the developing embryo, including both cytogenetic and metabolic/functional aspects. We are convinced that our work paves the way towards the integration of protein abundance measurements into embryo assessment procedures for two key reasons: first, by characterizing a large number of embryonic proteins that are detectable by a minimally invasive technique, and, second, by showing that it is possible, with existing technology, to detect proteins in single embryo secretions. As well as describing a novel proteomic methodology of extraordinary sensitivity, with the possibility of providing a means of minimally invasive embryo assessment in the future, this work has also led to the creation of a scientifically important catalogue of proteins present in the human blastocoel. We believe that this will be of broad interest and may have relevance within diverse fields of research.

On the biological side, I think that the authors should capitalize more on the characterization of the blastocoel proteome, which is a first, and the relationship between protein levels and aneuploidy, rather than on the implications for screening.

Comments and questions:

1- In page 5, some of the identified proteins were not validated by microarrays. The authors assume that these proteins are contaminants. While this could be the case, the

authors do not rule out the possibility of the persistence of proteins of maternal origin. Could the authors discuss how likely this is the case? It would also be interesting to see if any of those proteins is a good predictor of aneuploidy, and thus implicate maternal contributions in the success of IVF.

We strongly agree with the Reviewer on this point and have therefore introduced a paragraph where we discuss the possibility of endurance at the blastocyst stage of proteins synthetized prior to embryonic genome activation.
In order to provide documentation regarding the origin of unconfirmed proteins, we checked the literature for a human oocyte transcripts catalogue to compare with the list of detected blastosol proteins not validated by microarray.
Although several authors have investigated the transcriptome of human oocytes, none of the articles reviewed provides a comprehensive catalogue of transcripts.
Some of these studies present only a list of differentially expressed genes between the oocyte and another tissue (Vassena et al, 2011; Gayle et al, 2008), whilst others show partial subsets of genes of particular interest (Jaroudi et al, 2009; Bermúdez, 2004) or agglomerated data of gene ontology annotations (Wells et al, 2007).
The most comprehensive list of gene products we could find accounts for 564 transcripts highly active in human oocytes (Kocabas et al, 2006). We cross-checked our unconfirmed proteins with this list, identifying 1 protein out of 110 present in both catalogues. This was PSMA7 (Proteasome subunit alpha type-7) and it has now been referenced in section **Gene expression analysis in embryos** (line 152, page 6) where unconfirmed identifications are discussed. Kocabas article was also included in the bibliography. Due to the limited data available, it was not possible to establish a correlation between proteins of maternal origin and ploidy, thus precluding any speculation regarding the maternal contribution in the outcome of IVF based on the persistence of maternal effect proteins in blastocyst.

Vassena, R., Boué, S., González-Roca, E., Aran, B., Auer, H., Veiga, A., & Izpisua Belmonte, J. C. (2011). Waves of early transcriptional activation and pluripotency program initiation during human preimplantation development. *Development (Cambridge, England)*, *138*(17), 3699–709. doi:10.1242/dev.064741

Jones, G. M., Cram, D. S., Song, B., Magli, M. C., Gianaroli, L., Lacham-Kaplan, O., … Trounson, A. O. (2008). Gene expression profiling of human oocytes following in vivo or in vitro maturation. *Human Reproduction (Oxford, England)*, *23*(5), 1138–44. doi:10.1093/humrep/den085

Bermúdez, M. G., Wells, D., Malter, H., Munné, S., Cohen, J., & Steuerwald, N. M. (2004). Expression profiles of individual human oocytes using microarray technology. *Reproductive BioMedicine Online*, *8*(3), 325–337. doi:10.1016/S1472-6483(10)60913-3

Wells, D., & Patrizio, P. (2008). Gene expression profiling of human oocytes at different maturational stages and after in vitro maturation. *American Journal of Obstetrics and Gynecology*, *198*(4), 455.e1–9; discussion 455.e9–11. doi:10.1016/j.ajog.2007.12.030

Kocabas, A. M., Crosby, J., Ross, P. J., Otu, H. H., Beyhan, Z., Can, H., … Cibelli, J. B. (2006). The transcriptome of human oocytes. *Proceedings of the National Academy of Sciences of the United States of America*, *103*(38), 14027–32. doi:10.1073/pnas.0603227103

Jaroudi, S., Kakourou, G., Cawood, S., Doshi, A., Ranieri, D. M., Serhal, P., … SenGupta, S. B. (2009). Expression profiling of DNA repair genes in human oocytes and blastocysts using microarrays. *Human Reproduction (Oxford, England)*, *24*(10), 2649–55. doi:10.1093/humrep/dep224

2- On similar lines, since it has been shown that poor quality sperm could affect preimplantation embryo development (Janny and Menezo, 2005), I wonder whether data about sperm quality is available (sperm count, motility, and morphology), and whether this correlates with aneuploidy or any of the measured protein levels. This could be included in Table S8 (which, by the way, is not referenced in the manuscript). I realize that the sample size is inadequate for statistical analyses, but I think that this information, if available, is interesting to be reported.

SAMPLES	Morph score	Karyotype	Sex	Patient age	Semen parameters (Count, motility)	Insemination method	GAPDH (Log10 attomoles)	ACTA (Log10 attomoles)	H2A family (Log10 attomoles)	Euploid	H2A family detected
S01	4Dc	46 XY -1q	M	35	100 M, 54%	IVF	4.226	3.744	3.330	N	Y
S02	4Cb	46 XX	F	31	4 M, 28%	ICSI	3.979	2.984	-	Y	N
S03	4Dc	45 XX	F	36	127 M, 67%	IVF	3.769	3.050	3.817	N	Y
S04	4Db	49 XY	M	31	38 M, 59%	ICSI	3.675	2.763	3.324	N	Y
S05	3Bc	46 XX	F	36	7 M, 40%	ICSI	3.591	2.639	-	Y	N
S06	4Dc	48 XX	F	36	127 M, 67%	IVF	3.483	2.419	3.588	N	Y
S07	4Db	45 XO	Turner	36	7 M, 40%	ICSI	3.355	2.500	3.051	N	Y
S08	4Dc	46 XX	F	31	4 M, 28%	ICSI	3.336	2.330	-	Y	N
S09	4Bc	46 XX	F	31	4 M, 28%	ICSI	3.307	3.017	2.697	Y	Y
S10	4Bb	46 XY	M	36	127 M, 67%	IVF	3.294	2.198	-	Y	N
S11	4Bc	46 XX	F	30	0.5 M, 11%	ICSI	3.276	2.379	-	Y	N
S12	4Db	46 XY	M	36	127 M, 67%	IVF	3.181	3.050	3.683	Y	Y
S13	4Cc	46 XY	M	33	0.5 M, 11%	ICSI	2.723	2.896	-	Y	N
S14	3Dc	46 XY	M	30	107 M, 67%	IVF	2.476	2.097	3.108	Y	Y

Table EV1

Following the Reviewer's suggestion, we retrieved the data on semen count and motility used for the generation of the embryos analysed (morphology is not tested on the day of insemination and comments are only made if grossly abnormal morphology is detected). Semen count (in Million/ml) and motility (expressed as percentage of spermatozoa with progressive motility a+b, according to WHO Manual for Examination and processing of human semen) were added together with type of insemination performed (IVF, conventional in vitro fertilisation insemination; ICSI, intracytoplasmic sperm injection). We also carried out statistical analysis for the correlation of both semen parameters and insemination type with protein abundance however, as the Reviewer anticipated, we found no significance. We reported the raw data on **Table EV1** and referred to it in the main text, and included the statistical analysis on a new **Table EV2**.

(*Examination and processing of human semen*. (2010). *WHO laboratory manual* (Vol. Fifth Edit). World Health Organization.)

3- There is little overlap between the Urea-based preparation and Monoprep samples in terms of identified proteins (I had to calculate it, and it is 31 if I understood well). How many of those were validated by microarrays? Are they more abundant than others? Are they more likely to be contaminants? Some simple statistics could be performed to answer these questions.

We agree with the Reviewer regarding the lack of transparency in this set of data and have now introduced a new figure (**Figure 2A**, please see Point 3 in Review 1 section) where protein identification counts are shown per type of preparation.
We also performed some statistical analysis on these data as suggested by the Reviewer.

Data analyzed	All	Shared
Confirmed	182	21
Unconfirmed	106	10
Total	288	31
	P-value 0.697	

We compared the composition (confirmed/unconfirmed) of the list of proteins identified by both preparation procedures (Urea/Monoprep) and in total. Fisher's test shows no difference (P-value 0.697) in the composition of the groups (All= all protein identified, Shared= proteins present both in Urea and MonoPrep groups), suggesting that shared proteins are not significantly more confirmed by corresponding transcript detection than those proteins identified by one technique only.

A statistical analysis of protein abundance distributions showed that shared proteins (identified by both procedures) were not generally more abundant than those identified by a single method. Using parametric t-test, we did not find a statistical difference between the following comparison groups: Confirmed shared vs. Confirmed Urea only and Confirmed shared vs. Confirmed MonoPrep only. We may conclude that proteins identified by both MonoPrep and Urea preparations are not statistically more abundant than those identified by one technique only.

Urea	**Confirmed proteins**	**Abundance score** (Intensity/Mw) **Mean ± SEM, n**
	Shared with MonoPrep	3.564 ± 0.181, 21
	Urea only	3.566 ± 0.104, 80
	P-value	0.99

MonoPrep	**Confirmed proteins**	**Abundance score** (Intensity/Mw) **Mean ± SEM, n**
	Shared with Urea	2.207 ± 0.182, 21
	MonoPrep only	2.033 ± 0.067, 123
	P-value	0.33

This analysis was introduced in the **Gene expression analysis of embryos** section (line 144,

page 6) and the statistical methods applied are described in dedicated paragraph (line 619, page 23).

4- The authors report the intensities of protein targets measured in single blastocoels in Table S6. With the exception of GAPDH, and as already mentioned earlier, none of the proteins is quantified in all samples. There are actually more missing points than values. The authors do not discuss whether this is due to technical limitations, to fluctuations in the protein levels between different embryos (where in some embryos they are below the detection limits), or to the absence of the protein in some samples. I guess from the trend in figure 3a, one could argue that proteins with missing values are of lower abundance, and therefore technical limitations are to blame.

We believe the Reviewer has a valid point and in the reviewed version of the manuscript we set out to discuss this aspect in more depth.
We believe that the high rate of missing data points could be due to a combination of factors. Firstly, the measurement in single blastosols of some of the selected candidates revealed their abundance at the limit of detection of the targeted proteomics device used in this study. This reflected on missed detection of these targets in most of the samples and therefore, as the Reviewer suggested, this is potentially due to technical limitations. To corroborate this hypothesis we included a figure (**Figure 3A**) that shows targets abundance as detected by shotgun proteomics and their position in the total detection spectrum. As expected, targets less often identified are lower in the abundance ranking.
Alternatively, the lack of sensitivity for some of the candidates could be attributed to the use of SRMs based on peptides that do not possess biophysical characteristics that favour their detection.
On the other hand, for some of the targets a biological reason may be the cause of the missing value. For instance, H2A histone was detected in the blastosol of all aneuploid embryos, whilst the majority of blastosols derived from euploid ones did not show its presence. Although statistical significance was not reached, potentially due to the population small size, the data show a trend that may reflect a true biological difference.

These points are now discussed in the Discussion section (line 329, page 12) of the manuscript.

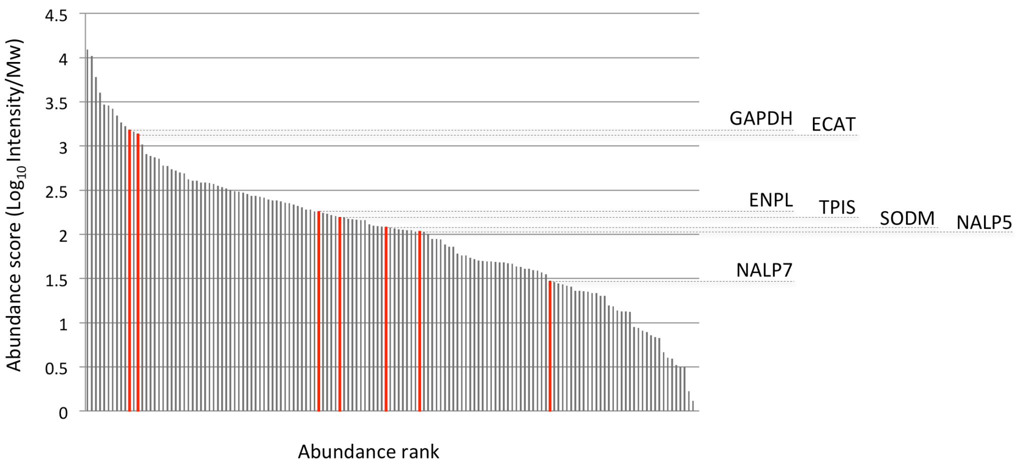

Figure 3A

5- Typically, it is good practice to choose at least two proteotypic peptides per protein or protein group to check for the stability of the SRM assay. One would like to see that the peptides belonging to the same protein correlate with each other. It is not clear to me why the authors have not done this and they should do so if they want to increase the robustness of their results.

We agree with the Reviewer that it is common practice in targeted proteomics to utilize more than one peptide per target protein. However, in this study, considering the challenging nature of the sample and in order to maximize the success rate of SRM assay development, we selected only peptides that were detected in the discovery phase with high precursor ion intensities. This greatly limited the number of peptides available for each protein since we also had to exclude miscleaved peptides and peptides containing methionines that do not yield reproducible quantification due to variable levels of oxidation. Additionally, we would like to stress that we used 5 transitions per peptide (independent signals from the same peptides) and that all our targeted proteomics assays were validated using synthetic peptides (**Figure EV1**).

6- I do not understand how the authors arrived to their final logistic regression model using the presence of H2A and abundance of GAPDH as predictors. How does each predictor perform alone? And how about other combinations? How do the different protein levels correlate to each other, and do aneuploid embryos have a different "signature" of those 9 proteins? I am not sure that this is possible given the missingness in the SRM data.

We have now included full details on statistical test for each individual parameter and protein detected. Also, we realized that details on the selection of parameters to use in the logistic regression analysis were missing and have now been introduced. Please refer to Point 2 of Reviewer 1 reply.

7- If the results of the authors have true biological meaning, then one would expect other

histones to also correlate with aneuploidy (thus in similar fashion as H2A). Given that histone H4 was one of the most abundant proteins identified in the monoprep procedure, it should be relatively straightforward to also examine the correlation between H4 abundance and aneuploidy. It is unclear why the authors chose not to do this and why they selected histone H2A and not H4 for the SRM measurements?

We welcome the Reviewer's valid comment on this topic and would like to explain why we used only one histone SRM assay. Initially, due to the minute size of the sample and the extremely low abundance of proteins within, Histones SRM was performed to provide a possible positive control. Only retrospectively it provided valuable information regarding embryo competence status. In future studies we will be able to include other histones as additional data points to test the presence of nuclear proteins in the blastocoel. We now mention this on line 230, page 9.

8- The authors do not discuss how the technique could be possibly improved to increase sensitivity.

This is a fair point and we now discuss limitations and future improvements to the technique in association with the arguments of Reviewer 3 Point 4 (line 338, page 13 and line 365, page 14).

"A major challenge of this methodology is the sensitivity required to detect proteins present in the minute blastosol volume. In fact, among the proteins tested, only those with higher abundance were regularly detected in the specimen (Figure 3B). This limited sensitivity could be attributed both to technical and biological aspects. As shown in Figure 3A, some of the proteins that were detected only in a subset of samples show lower than average concentrations, as determined from their intensities in shotgun proteomic experiments. The reproducible quantification of these targets is more challenging since their concentration is close to the limit of detection of our targeted proteomic setup. However, the inconsistent detection of some targets, as in the case of histones proteins (H2A), could be attributed to a biological variability, possibly associated to deviant physiological conditions. Increased detectability of targets should be addressed in the future by targeting alternative peptides, further optimizing procedures to minimize sample loss and utilizing alternative investigative devices, including both more sensitive mass spectrometers and digital immunoassays."

"However, due to the small size of the sample population investigated, the limited amount of predictors used and the retrospective nature of the analysis, these data need to be treated cautiously. It is essential to generate additional data in larger studies in order to test the validity of this proteomic approach to preimplantation aneuploidy detection and to define the real sensitivity and specificity of this methodology. Also, targeted mass spectrometry represented the most sensitive technology that allowed us to design assays for a number of target proteins without the need for specific reagents (e.g. antibodies). In the future, alternative novel methodologies (i.e. single molecule arrays, digital ELISA) could be implemented to improve the sensitivity and comprehensiveness of human blastosol profiling in single embryos."

Minor comments:

The authors do not refer to figure 3b until the discussion, but it is covered earlier in the text (specifically on p8)

All figures are now correctly referred throughout the text.

In p7, authors refer to Glyceraldehyde 3-phosphate dehydrogenase as G3P, but in other places as GAPDH.

Noted and addressed.

In the materials and methods, the statistical analysis section has very little information about the logistic regression model used and the cross validation.

In order to increase transparency we now provide a detailed explanation of the logistic regression model as well as other statistical tests employed in the manuscript (starting from line 230, page 9) and the material and methods "**Statistics**" section (line 619, page 23).

2nd Editorial Decision 20 August 2015

Thank you for the submission of your revised manuscript to EMBO Molecular Medicine. We have now received the enclosed reports from the referees that were asked to re-assess it. As you will see the reviewers are now globally supportive and I am pleased to inform you that we will be able to accept your manuscript pending the following final amendments:

Please address the minor text change commented by referee 1 point 2. Make sure that the limitations mentioned in point 1 are adequately discussed. Please provide a letter INCLUDING the reviewer's reports and your detailed responses to their comments (as Word file).

I look forward to reading a new revised version of your manuscript within 2 weeks.

***** Reviewer's comments *****

Referee #1 (Comments on Novelty/Model System):

The study is performed by an outstanding team of investigators, it is well written, uses state of the art techniques and it is technically sound. The use of human embryos makes it highly valuable and worth of publication.

Referee #1 (Remarks):

The resubmission by Poli et al addresses many of the questions sent in the first submission. The paper overall is excellent and it uses state of the art techniques.
- However, the ploidy prediction component of the paper using GAPDH, actin and H2A remains unsatisfactory and should be removed. Again, the low number of samples tested and the use of only 3 proteins to make prediction is not acceptable.
- Further the method description of the microarray data raises more questions: in the method section it is stated that 3 pooled samples (from 3,3 and 4 embryos) were separated in 3 ICM and 3 TE (therefore 3 replicates) and then amplified.
1. How were the ICM and TE of the embryo separated?
2. Why does the result section describe whole embryos (line 125) results and not TE/ ICM?
3. Please clarify what is the reference human genome used to compare the embryo samples and how did they do the comparison? If TE and ICM RNA was obtained, the comparison should be done between the 3 TE samples to the 3 ICM samples, like done in Adjaye et al (2005) for human embryos (and by others in rodents). The analysis should be repeated in this fashion.

Referee #3 (Comments on Novelty/Model System):

Please see summarizing text below.

Referee #3 (Remarks):

In their revised version of their manuscript and response to authors, Polli and colleagues considerably improve the clarity of the text. Specifically, I am satisfied with their reply to our and other referees' comments concerning statistical details and gene list overlaps. On the technical side, however, the authors do not introduce any new supporting data, in spite of our request, but decided to settle the outstanding issues by simply acknowledging the shortcomings of their approach. In addition, they discuss future approaches and improvements as requested. I still believe therefore that the paper in its revised form is somewhat falling short in terms of data size and quality (due to low sample size and technical limitations), but the field may nevertheless perceive this paper as an interesting first step toward the comprehensive, proteomic profiling of human blastocysts.

Referee #1 (Comments on Novelty/Model System):

The study is performed by an outstanding team of investigators, it is well written, uses state of the art techniques and it is technically sound. The use of human embryos makes it highly valuable and worth of publication.

Referee #1 (Remarks):

The resubmission by Poli et al addresses many of the questions sent in the first submission. The paper overall is excellent and it uses state of the art techniques.

However, the ploidy prediction component of the paper using GAPDH, actin and H2A remains unsatisfactory and should be removed. Again, the low number of samples tested and the use of only 3 proteins to make prediction is not acceptable.

Further the method description of the microarray data raises more questions: in the method section it is stated that 3 pooled samples (from 3, 3 and 4 embryos) were separated in 3 ICM and 3 TE (therefore 3 replicates) and then amplified.

1. How were the ICM and TE of the embryo separated?

We now include a brief description of the ICM cells excision in the main text (line 131 page 5) and a detailed one in the Material and methods section (line 563, page 21).

2. Why does the result section describe whole embryos (line 125) results and not TE/ ICM?

Our gene expression analysis showed that 80% of the genes were expressed by both ICM and TE. Additionally, differential expression analysis performed on the transcripts expressed by both ICM and TE did not return any significant case in our dataset. For these reasons, we combined the lists of actively transcribed genes in the two tissue groups into a unified, comprehensive catalogue of genes actively expressed by the human blastocyst. This catalogue was then used to confirm the embryonic origin of the blastocoelic proteins identified with mass spectrometry experiments. Additionally to the text implementations in the manuscript discussed above, we have now included a Venn diagram in **Figure EV2A** to clarify the origin of the transcripts detected.

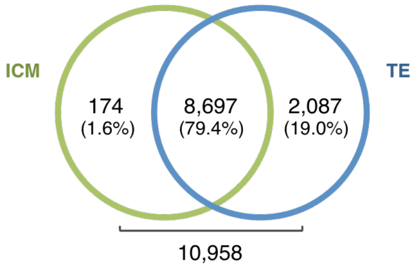

Figure EV2A

3. Please clarify what is the reference human genome used to compare the embryo samples and how did they do the comparison? If TE and ICM RNA was obtained, the comparison should be done between the 3 TE samples to the 3 ICM samples, like done in Adjaye et al (2005) for human embryos (and by others in rodents). The analysis should be repeated in this fashion.

Since we did not identify significant differences in the gene expression profiles of the ICM and TE, as explained in the reply to the previous point, we feel that, with the current data, the comparison of the tissues would not be very informative. For these reasons, we decided to maintain the comparison between the human blastocyst expressed genes and the Panther database reference human genome. To improve clarity of this analysis, we have included in Figure EV2B legend the following sentence:

"Blastocyst gene expression investigated using Panther database statistical overrepresentation test comparing the global blastocyst transcript list to the default human whole genome list, which included all genes present in the Panther database."

Referee #3 (Comments on Novelty/Model System):

Please see summarizing text below.

Referee #3 (Remarks):

In their revised version of their manuscript and response to authors, Poli and colleagues considerably improve the clarity of the text. Specifically, I am satisfied with their reply to our and other referees' comments concerning statistical details and gene list overlaps. On the technical side, however, the authors do not introduce any new supporting data, in spite of our request, but decided to settle the outstanding issues by simply acknowledging the shortcomings of their approach. In addition, they discuss future approaches and improvements as requested. I still believe therefore that the paper in its revised form is somewhat falling short in terms of data size and quality (due to low sample size and technical limitations), but the field may nevertheless perceive this paper as an interesting first step toward the comprehensive, proteomic profiling of human blastocysts.
